# Synbiotic supplementation modulates humoral immunity and cecal microbiota in broiler chickens exposed to subclinical doses of fumonisins and deoxynivalenol

**DOI:** 10.3389/fphys.2026.1808482

**Published:** 2026-04-21

**Authors:** Joseph Rishitha Dasireddy, Laharika Kappari, Ramesh K. Selvaraj, Todd J. Applegate, Revathi Shanmugasundaram

**Affiliations:** 1 Department of Poultry Science, University of Georgia, Athens, GA, United States; 2 Toxicology and Mycotoxin Research Unit, United States Department of Agriculture - Agricultural Research Service (USDA-ARS), Athens, GA, United States

**Keywords:** Deoxynivalenol, fumonisin, microbiota, SCFA, synbiotic

## Abstract

Synbiotics modulate the cecal microbiota in chickens by promoting the growth of beneficial bacteria, which have the potential to mitigate the negative effects of mycotoxins such as fumonisins (FUM) and deoxynivalenol (DON) and improve immune responses. The objective of the study was to evaluate the effect of synbiotic supplementation (0.05%) on the bile IgA, serum IgY, cecal microbiota composition, diversity, and short-chain fatty acid (SCFA) profile in broilers exposed to subclinical concentrations of FUM and DON. A total of 360 one-day-old broilers were distributed into 4 treatments: Control, Mycotoxin (8.5 FUM + 3.8 DON mg/kg diet), Synbiotic, and Mycotoxin + Synbiotic. Cecal contents were collected on d21 and d35, and bacterial compositions were identified by analyzing the V3–V4 region of the 16S rRNA gene using Illumina sequencing. Relative abundance of families and alpha diversity indices were analyzed using the Kruskal-Wallis H Test, and IgA, IgY, and short-chain fatty acids (SCFAs) were analyzed using two-way ANOVA. On d35, there were no interaction effects between FUM + DON and synbiotic supplementation on bile FUM-specific IgA concentrations and serum FUM and DON-specific IgY concentrations (*p* > 0.05). There were no significant interactions between FUM + DON and synbiotic supplementation on the cecal SCFA levels on d21 and d35 (*p* > 0.05). But on d35, there was a trend in the main effect of FUM + DON on the cecal propionate concentration. Propionate concentration was decreased by 36.4% compared to the no mycotoxin treatment groups (*p* = 0.09). There were no significant differences in the Shannon diversity index between treatment groups (*p* > 0.05). On d35, FUM + DON increased the ratio of relative abundances of Firmicutes to Bacteroidetes in the treatment groups, while synbiotic supplementation further increased the Firmicutes to Bacteroidetes ratio in the FUM + DON treatment group. In conclusion, dietary supplementation of synbiotics at 0.05% has supported mucosal immunity and altered cecal microbial composition and fermentation profiles in broilers exposed to subclinical FUM + DON, without affecting overall microbial diversity.

## 1 Introduction

Mycotoxins are toxic secondary metabolites produced by various molds, including *Aspergillus*, *Fusarium*, and *Penicillium* species (Tola and Kebede, 2016). Chronic ingestion of low levels of mycotoxin-contaminated feed can lead to metabolic and physiologic disturbances in chickens (Bryden, 2012). In North America, fumonisins (FUM) and deoxynivalenol (DON) are among the most prevalent mycotoxins detected in poultry feed ingredients (dsm-firmenich, 2024). A healthy gut in broilers is characterized by intact tight junctions and a balanced microbial community, which collectively promote efficient nutrient absorption and minimize immune system activation (Qamar et al., 2021; Wickramasuriya et al., 2022). However, exposure to mycotoxins disrupts the tightly regulated intestinal environment. Due to their relatively low intestinal absorption rates, FUM and DON remain in the gut lumen for extended periods, resulting in an increase in their interaction with intestinal epithelial cells. Consequently, intestinal epithelial cells are exposed to higher concentrations of these toxins, leading to impaired intestinal function and leaky gut (Ren et al., 2019; Yakout, 2024). Disruption of gut epithelial integrity alters the gut microbiota composition, ultimately compromising overall health and immune competence in chickens (Antonissen et al., 2015a; Paraskeuas et al., 2021; Shanmugasundaram et al., 2023).

Gut microbes play a critical role in digestion, nutrient absorption, contribute to energy supply and regulate the physiological homeostasis (Duca and Lam, 2014). The gut microbial community also directly influences host immune system development and function (Kogut and Arsenault, 2016). Previous studies reported that combined dietary exposure to 3 mg FUM + 4 mg DON per kg diet alters cecal microbiota composition by decreasing the *Lactobacillus* population, microbial diversity and richness in chickens (Antonissen et al., 2015a; Shanmugasundaram et al., 2023). Exposure to subclinical doses of Fusarium toxins has also been associated with decreased mucosal antibody responses in chickens (Shanmugasundaram et al., 2025, Shanmugasundaram et al., 2023). In particular, DON-contamination alone (10mg/kg diet) decreased antibody response to infectious bronchitis vaccine (Ghareeb et al., 2012) and Newcastle disease virus (Danicke et al., 2002) in chickens. The gut microbiota and the avian immune function are an interconnected axis that supports intestinal homeostasis. Microbial communities modulate epithelial barrier integrity, shape immune cell activity, and influence antibody production through microbe−associated molecular patterns and metabolite driven pathways (Yoo et al., 2020). Among the microbiota-derived metabolites, SCFAs play a crucial role in maintaining intestinal homeostasis and immune function (Wang et al., 2023), thereby promoting gut epithelial integrity and strengthening the host’s immune response (Oke et al., 2025). Mycotoxin exposure disrupts this axis by reducing beneficial taxa, lowering short−chain fatty acids, and mucosal antibody responses. Probiotic supplementation has also been shown to counteract these mycotoxin-induced dysbiosis by improving IgA levels and increasing beneficial bacterial taxa (Muhammad et al., 2026).

Lactic acid bacteria, such as *Lactobacillus* and *Bifidobacterium* spp., can adsorb mycotoxins such as aflatoxin, patulin, and ochratoxin via interactions with the peptidoglycan layer of their cell wall (Azevedo and Gierus, 2025; Fuchs et al., 2008; Peltonen et al., 2001). This results in decreased mycotoxin adhesion to the gut epithelium and helps maintain gut integrity and microbial homeostasis (Jeong et al., 2024). Dietary supplementation with probiotics, prebiotics, and synbiotics has shown beneficial effects in modulating intestinal microbiota and mitigating mycotoxin-induced gut damage (McCormick, 2013; Schatzmayr et al., 2006; Śliżewska et al., 2020). For example, supplementation with *L. casei* and *Candida utilis* for 42 days reversed jejunal reductions in *L. aviarius* and *Bacillus subtilis* caused by combined aflatoxin B1 (AFB1) (14 μg/kg diet) and zearalenone (ZEA) (57 μg/kg diet), and had a positive correlation with improved production performance (Chang et al., 2020). Similarly, a mycotoxin detoxifier (1g/kg) containing *Enterococcus faecium*, montmorillonite, and AFB1-degrading enzyme from *Aspergillus oryzae* decreased pathogenic *E. coli, Shigella, and Staphylococcus* in broilers fed AFB1 (40 μg/kg diet) contaminated diets (Guo et al., 2023).

Although numerous studies have demonstrated the efficacy of probiotics and prebiotics in detoxifying mycotoxins *in vitro*, research on *in vivo* synbiotic supplementation under multiple mycotoxin exposure in broilers remains limited. In our previous study with synbiotics containing *B. animalis, P. acidilactici, E. faecium*, along with fructo-oligosaccharides (0.05%) reversed the negative effects of combined FUM (8.5 mg/kg) and DON (3.9 mg/kg) on growth performance, jejunal and ileal villus length and crypt depth, tight junction protein expression, and CD8^+^:CD4^+^ T cells ratio (Dasireddy et al., 2025). Importantly, we also observed a significant decrease in *Lactobacillus* populations in birds exposed to FUM + DON. Alterations in gut morphology and barrier integrity caused by FUM + DON promote colonization by pathogenic bacteria and disrupt intestinal microbial composition, which can be ameliorated through synbiotic supplementation (Antonissen et al., 2015a; Shanmugasundaram et al., 2023). However, limited information exists on gut microbiota changes in response to combined subclinical doses of FUM and DON. The current study is a follow−up of the previously published paper from the same experimental trial (Dasireddy et al., 2025), which focused primarily on production performance, cell−mediated immunity, including cytokine expression and immune cell population dynamics. The current study builds on those findings as a follow−up investigation, with emphasis on humoral immune responses and gut microbiome dynamics to provide a more comprehensive evaluation of host responses to combined mycotoxin exposure and synbiotic intervention. Based on previous findings, we hypothesized that synbiotic supplementation mitigates FUM + DON induced gut dysbiosis by promoting beneficial taxa and their metabolic activity, thereby improving SCFA production, and supporting the mucosal antibody responses in broilers. Therefore, the objective of this study was to evaluate the effects of combined dietary exposure to 8.5 mg/kg FUM + 3.9 mg/kg DON on the gut microbiome and to determine whether synbiotic supplementation could alleviate mycotoxin-induced damage, thereby explaining the mechanism underlying improved production performance.

## 2 Materials and methods

### 2.1 Experimental diet formulation

A non-medicated corn-soybean meal-based mash diet was used as a basal diet and divided into starter (d0-21) and grower (d22-35) phases (**Table 1**). FUM and DON were produced on rice cultures separately using *F. verticillioides* M-3125 and *F. graminearum* DSM-4528, as described earlier (Liu J. et al., 2023). The rice cultures were homogenized and premixed with a small portion of the basal diet, then blended with the remaining feed to prepare uniform experimental diets. The synbiotic used in this study was a commercial proprietary formulation (DSM firmenich), containing probiotic strains (*Bifidobacterium animalis*, *Pediococcus acidilactici*, and *Enterococcus faecium*) with fructooligosaccharides. The synbiotic was incorporated into the diet at 0.05% according to the manufacturer’s recommendation. LC-MS-MS was used to determine the final concentration of mycotoxins in the finished diets (Romer Labs, Union, MO, United States; **Table 2**).

**Table 1:** Ingredient and nutrient composition of the basal diet (as-fed basis).

Ingredient	Starter (%)	Finisher (%)
Corn	56.29	64.86
Soybean meal, 48% CP	37.87	28.44
Soybean oil	2.18	3.80
Dicalcium phosphate	1.48	0.84
Calcium carbonate	0.91	0.78
Sodium chloride	0.40	0.40
MHA	0.37	0.32
L-lysine	0.21	0.22
Trace mineral premix1	0.10	0.10
Choline chloride (60%)	0.07	0.08
L-threonine	0.06	0.07
Vitamin premix2	0.05	0.05
Phytase (500FTU)	0.01	0.01

^1^Supplied per kilogram of diet: Mn, 107.2 mg; Zn, 85.6 mg; Mg, 21.44 mg; Fe, 21.04; Cu, 3.2 mg; I, 0.8 mg; Se, 0.32 mg. ^2^Supplied per kilogram of diet: vitamin A, 5,511 IU; vitamin D3, 1,102 ICU; vitamin E, 11.02 IU; vitamin B12, 0.01 mg; biotin, 0.11 mg; menadione, 1.1 mg; thiamine, 2.21 mg; riboflavin, 4.41 mg; d-pantothenic acid, 11.02 mg; vitamin B6, 2.21 mg; niacin, 44.09 mg; folic acid, 0.55 mg; choline, 191.36 mg.

**Table 2:** Analyzed mycotoxin content of experimental diets.

	Treatment	Total Fumonisins (FUM) (FB1+FB2+FB3) (mg/kg)	FB1 (mg/kg)	DON (mg/kg)	ZEA (mg/kg)	Total Mycotoxins (mg/kg)
Starter	T1 (Control)	2.5	1.8	1.1	0.08	5.6
	T2 (Mycotoxin)	8.7	6.2	3.8	1.3	18.6
	T3 (Synbiotic)	2.6	1.9	1.1	0.01	6.3
	T4 (Mycotoxin + Synbiotic)	8.5	6.3	4.2	1.6	23.0
Grower	T1 (Control)	2.6	1.9	0.9	0.07	4.7
	T2 (Mycotoxin)	8.5	6.2	3.8	0.6	16.8
	T3 (Synbiotic)	2.4	1.8	1.0	0.6	5.0
	T4 (Mycotoxin + Synbiotic)	8.6	6.2	3.9	0.5	17.0

Representative samples of feeds from treatments (T) 1 to 4 were analyzed by LC-MS-MS in Romer labs (Union, MO, USA) for fumonisin (FUM), deoxynivalenol (DON), zearalenone (ZEA), and total mycotoxins (including other metabolites like 15 acetyl DON, Type B Trichothecenes, and Aflatoxins) concentrations.8.5 mg/kg FUM and 3.9 mg/kg of DON; 0.05% synbiotic.

### 2.2 Study design

A 35-day feeding trial was conducted using a total of 360 one-day-old Cobb 500 male broiler chicks (from the Cobb-Vantress Hatchery in Cleveland, GA). Birds were vaccinated on day 0 against *Eimeria* using COCCIVAC^®^-B52 (Merck Animal Health, NJ, USA). All animal procedures were approved by the Institutional Animal Care and Use Committee at the Southern Poultry Research Group, Athens, GA (Protocol No. USM102023-117) and ARRIVE 2.0 guidelines. Chicks were housed in 5×5 feet floor pens with a stocking density of 1.0 ft^2^ per bird, on fresh wood shavings following standard North American industry practices. Each pen was equipped with one tube feeder and one drinker (15 birds per feeder/drinker ratio). Thermostatically controlled gas heaters were used as the primary heat source for the poultry house. Chicks had *ad libitum* access to feed and water throughout the experimental period, and mortality was recorded daily.

### 2.3 Experimental setup and sampling

**Figure 1:**
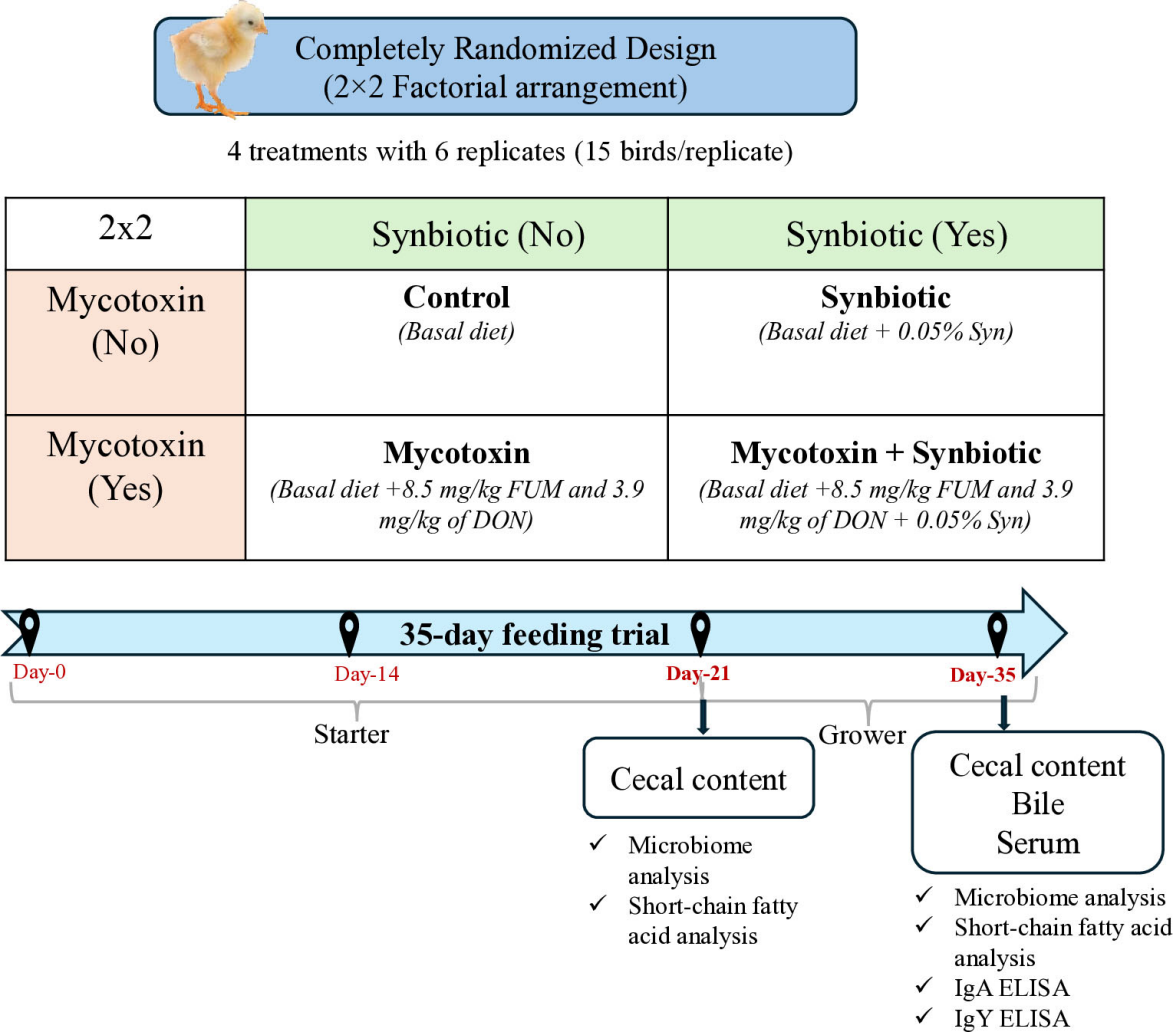
Schematic illustration of the experimental design. A total of 360 Cobb 500 broiler chicks were randomly assigned to four dietary treatments in a 2 × 2 factorial arrangement with two factors: mycotoxin (0 or 8.5 mg/kg fumonisins + 4 mg/kg deoxynivalenol) and synbiotic supplementation (0 or 0.05%). Each treatment had six replicate pens with 15 birds per pen. On days 21 and 35, one bird per pen was euthanized for sample collection. Cecal contents were collected for microbiome (16S rRNA sequencing) and SCFA analyses, and bile and serum were collected on day 35 for immunoglobulin analysis.

Birds were randomly allocated to four dietary treatments in a 2×2 factorial arrangement (n = 6) with six replicates per treatment (15 birds per pen) as illustrated in **Figure 1**. Treatment groups were assigned to pens using a randomized complete block design. Randomization and pen assignment were performed using random permutation tables following the procedures described by Cochran and Cox (1992). The experimental treatment groups were T1 - Control diet; T2 - Mycotoxin contaminated diet; T3 - Synbiotic supplemented diet; and T4 - Mycotoxin contaminated diet + Synbiotic supplementation. On days 21 and 35, one bird per pen was euthanized using CO_2_ at a flow rate of 30% of the chamber volume per minute until cessation of breathing and heartbeat, in accordance with the Institutional Animal Care and Use Committee at the Southern Poultry Research Group, Athens, GA.

### 2.4 Bile and serum FB1-specific and DON-specific IgA and IgY quantification by ELISA

On d35, bile and serum samples were collected from one bird per pen and stored at -20 °C until analysis. Anti-FB1 and anti-DON immunoglobulin A (IgA) in bile and IgY in serum were quantified using enzyme-linked immunosorbent assay (ELISA). Flat-bottomed, high-binding 96-well plates (Greiner Bio-One, Monroe, NC) were coated with 100 μL/well of 2.5 μg/mL FB1 or DON in 0.1 M carbonate buffer (pH 9.6) and incubated overnight at 4 °C. Plates were washed three times with wash buffer (0.05% Tween 20 in PBS, pH 7.4) and blocked with 200 μL/well of SuperBlock™ (PBS) Blocking Buffer (Thermo Fisher Scientific) for 1 h at 37 °C. Checkerboard titration was used to determine optimal sample dilutions. For IgA quantification, bile samples were diluted 1:100 for FB1-specific IgA and 1:50 for DON-specific IgA in SuperBlock™ (PBS) Blocking Buffer. For IgY quantification, serum samples were diluted 1:200 in blocking buffer. 100 μL of samples were added per well in duplicate and incubated for 1 h at room temperature. After washing, 100 μL/well of horseradish peroxidase-conjugated secondary antibody was added: anti-chicken IgA (Novus Biologicals, Littleton, CO, USA) for bile samples and goat anti-chicken IgY (H+L)-HRP (Southern Biotech, Birmingham, AL, USA) for serum samples, both diluted 1:100,000 in blocking buffer. Subsequently, 100 μL/well of 3, 3, 5, 5-tetramethylbenzidine (TMB) substrate (Sigma-Aldrich, St. Louis, MO) was added and incubated for 6 minutes. The reaction was stopped with 100 μL/well of 1 M HCl, and absorbance was measured at 450 nm using a Synergy HTX multimode microplate reader (BioTek, VT, USA). Antibody concentrations were reported as mean optical density values (Cason et al., 2023).

### 2.5 Cecal DNA isolation and 16S rRNA gene amplification

At the end of the starter (d21) and grower (d35) periods, cecal content samples were collected from each pen into sterile 15 mL tubes, immediately placed on ice, and stored at -20 °C until further analysis. Bacterial genomic DNA was extracted following a protocol as described earlier (Shanmugasundaram et al., 2019). Extracted DNA samples were sent to Kelly Products Inc. (Georgia, USA) for sequencing on an Illumina platform. The V3–V4 region of the bacterial 16S rRNA gene was amplified using the S-D-Bact-0341-b-S-17 (5′-CCTACGGGNGGCWGCAG-3′) forward and S-D-Bact-0785-a-A-21 (5′-GACTACHVGGGTATCTAATCC-3′) primer pairs as described (Klindworth et al., 2013).

### 2.6 Bioinformatics analysis

For microbiome data, 16S rRNA gene sequencing was performed on an Illumina MiSeq Platform using 250-bp paired-end reads. Raw sequencing data were converted into FASTQ files, and the paired-end sequences were imported into QIIME 2 (Bolyen et al., 2019) for processing. Quality filtering, denoising, paired-end merging and chimera removal were performed using the DADA2 plugin (Callahan et al., 2016). Quality filtering parameters followed DADA2 defaults unless otherwise specified. Low quality bases were trimmed where Phred quality dropped below 30, and chimeric sequences were removed using the consensus method. Across samples, the number of raw input reads ranged from 5,623 to 31,027. After quality filtering and chimera removal, 48-52% of reads were retained, resulting in 989–15,055 high-quality non-chimeric reads per sample. The final feature table contained 328,678 sequences across 48 samples, representing 959 amplicon sequence variants (ASVs). Representative sequence lengths ranged from 313 to 424 bp, with an average length of 402.6 ± 8.7 bp. Amplicons shorter than 313 bp or longer than 424 bp were excluded to remove non−specific or low−quality amplicons. The resulting ASVs were taxonomically classified using a pre−trained naïve Bayes classifier trained on the SILVA 138 SSU reference database (Quast et al., 2012). ASVs contributing less than 0.01% of the total relative abundance across all samples were removed prior to downstream community analyses.

Before diversity analyses, the feature table was rarefied to 989 reads per sample, corresponding to the lowest sequencing depth among all samples. This rarefaction depth ensured retention of all samples while standardizing sequencing depth across the dataset. Alpha diversity metrics included observed ASVs (richness), Shannon diversity, Faith’s phylogenetic diversity index, and the Chao−1 richness estimator. Beta diversity analyses were visualized using EMPeror (Vázquez-Baeza et al., 2013).

### 2.7 Short-chain fatty acid analyses

On d21 and d35, cecal content samples were collected and analyzed for short-chain fatty acids (SCFAs), including acetate, propionate, butyrate, valerate, isovalerate, and isobutyrate, using gas chromatography as described earlier (Lourenco et al., 2020). Briefly, samples were mixed with metaphosphoric acid solution (25% wt/vol) and extracted with ethyl acetate. SCFA concentrations were determined using a Shimadzu GC-2010 Plus (Shimadzu Corporation, Kyoto, Japan) gas chromatography equipped with a flame ionization detector and a Zebron ZB-FFAP capillary column (Phenomenex Inc., Torrance, CA, USA). The sample injection volume was 1.0 μL, and helium was used as the carrier gas. The column temperature was maintained at 110 °C, and injector and detector temperatures were set at 250 °C and 350 °C, respectively. SCFA concentrations were determined by comparing sample peak heights to those of actual standards.

### 2.8 Statistical analysis

A two-way ANOVA (JMP Pro 15 software, Cary, NC, USA) was performed to evaluate the interaction effects of subclinical doses of mycotoxin × synbiotic treatments on bile anti-FUM and anti-DON IgA, serum anti-FUM, anti-DON IgY, and SCFA, with the pen considered the experimental unit (n=6). When a significant interaction was detected (P < 0.05), means were separated using Tukey’s HSD. If no interaction was observed (*p* > 0.05), main effects were reported. Results with a *p-*value between 0.05 and 0.1 were reported as a trend. For microbiome data, differences in individual taxa and alpha diversity indices among treatments were analyzed using the Kruskal-Wallis H Test. To control for multiple comparisons and reduce the likelihood of false-positive findings, p−values were adjusted using the Benjamini–Hochberg false discovery rate (FDR) correction. FDR−adjusted p−values ≤ 0.05 were considered statistically significant, and results with 0.05 < *p* ≤ 0.10 were reported as a trend. Spearman correlation analyses were performed to evaluate the association between major microbial families and cecal SCFAs concentrations on d21 and d35. In addition, correlations between microbial phyla and host immune parameters (IgA and IgY) were assessed. *P*−values were corrected for multiple testing using the Benjamini–Hochberg FDR method. Functional pathway profiles were predicted from 16S rRNA gene sequences using PICRUSt2, and pathway abundances were annotated based on the MetaCyc database. Differences in predicted pathway abundances among treatments were assessed using the Kruskal-Wallis test, and *p*-values were adjusted for multiple comparisons using the FDR correction method.

## 3 Results

### 3.1 Effect of synbiotic supplementation on bile FUM-specific IgA antibody concentrations in broiler chickens fed mycotoxin-contaminated diets

**Figure 2:**
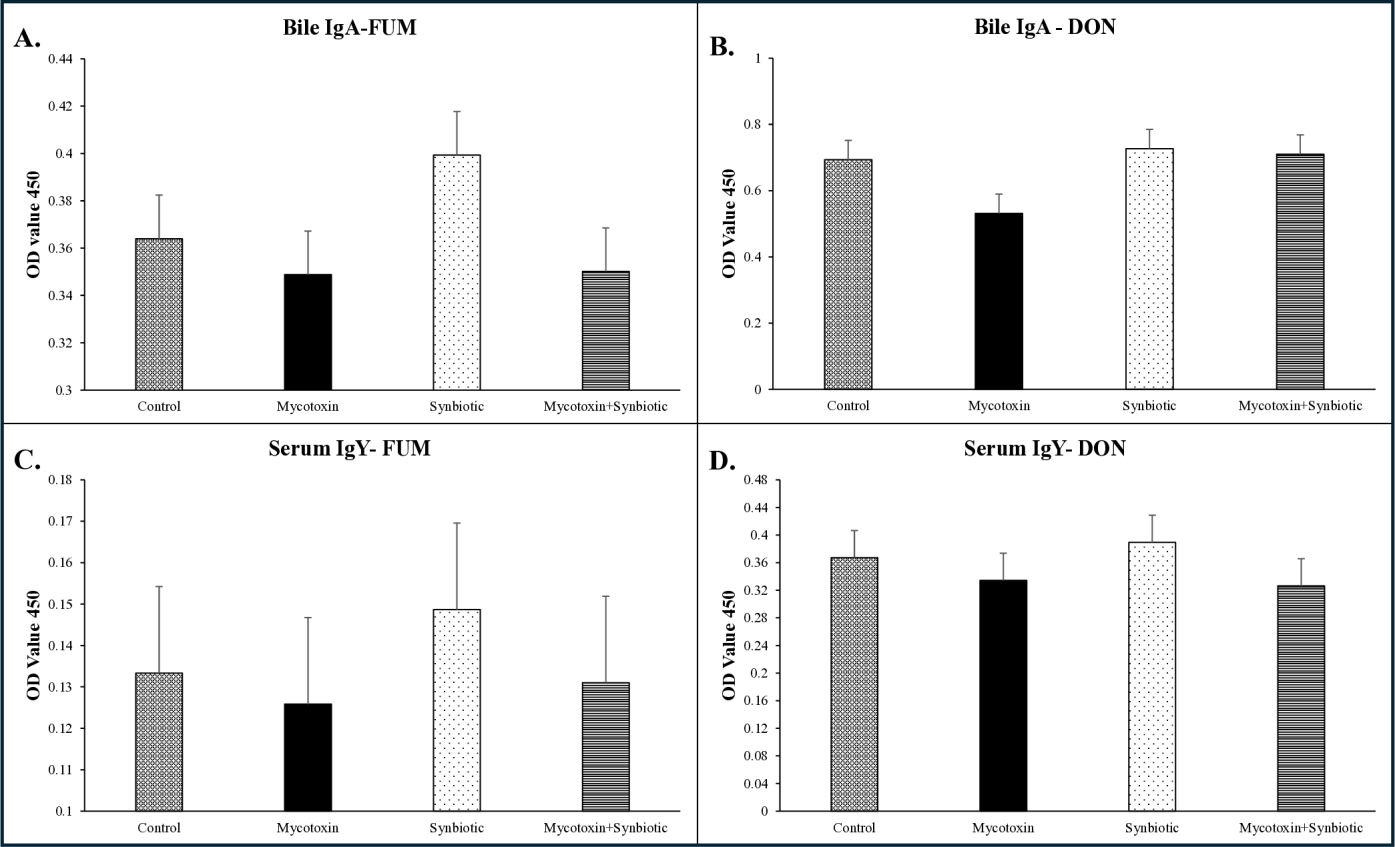
Effects of synbiotic supplementation on bile and serum FUM-specific IgA, IgY, and DON-specific IgA, IgY antibody concentrations in broiler chickens fed mycotoxin-contaminated diets. Birds were supplemented with 0.05% synbiotic from d0 to d35 (n = 6). Mycotoxin-contaminated diets (8.5 mg/kg FUM + 3.9 mg/kg DON) were fed from d0 to d35 in a 2 × 2 factorial arrangement. On d35, bile samples were analyzed for FUM-specific **(A)** and DON-specific **(B)** IgA antibodies, and serum samples were analyzed for FUM-specific **(C)** and DON-specific **(D)** IgY antibodies, expressed as optical density values. Bars represent means ± SEM. Bars within each panel **(A–D)** with no common superscripts differ significantly (P < 0.05). P-values: **(A)** FUM-specific IgA: mycotoxin × synbiotic = 0.36; mycotoxin = 0.09; synbiotic = 0.33. **(B)** DON-specific IgA: mycotoxin × synbiotic = 0.22; mycotoxin = 0.14; synbiotic = 0.08. **(C)** FUM-specific IgY: mycotoxin × synbiotic = 0.82; mycotoxin = 0.55; synbiotic = 0.70. **(D)** DON-specific IgY: mycotoxin × synbiotic = 0.70; mycotoxin = 0.24; synbiotic = 0.86.f

On d35, there were no significant interactions or main effects of FUM + DON, and synbiotic supplementation was observed on bile FUM-specific IgA concentrations among treatment groups (*p* > 0.05) (**Figure 2A**). However, a trend was observed for the main effect of FUM + DON (*p* = 0.09), where FUM + DON decreased bile FUM-specific IgA concentrations by 8.4% compared with the no-mycotoxin treatment groups.

### 3.2 Effect of synbiotic supplementation on bile DON-specific IgA antibody concentrations in broiler chickens fed mycotoxin-contaminated diets

On d35, no significant interactions or main effects were detected for bile DON-specific IgA concentrations (*p* > 0.05) (**Figure 2B**). However, a trend was observed for the main effect of synbiotic supplementation (*p* = 0.08), where synbiotic supplementation increased bile DON-specific IgA titers by 17.4% compared to no synbiotic treatment groups.

### 3.3 Effect of synbiotic supplementation on serum FUM-specific IgY antibody concentrations in broiler chickens fed mycotoxin-contaminated diets

On d35, serum FUM-specific IgY concentrations were not significantly affected by FUM + DON or synbiotic supplementation (*p* > 0.05) (**Figure 2C**).

### 3.4 Effect of synbiotic supplementation on serum DON-specific IgY antibody concentrations in broiler chickens fed mycotoxin-contaminated diets

On d35, serum DON-specific IgY concentrations showed no significant interactions or main effects between FUM + DON and synbiotic supplementation among treatment groups (*p* > 0.05) (**Figure 2D**).

### 3.5 Effect of synbiotic supplementation on the short-chain fatty acid concentrations of cecal contents of broiler chickens fed mycotoxin-contaminated diets

**Figure 3:**
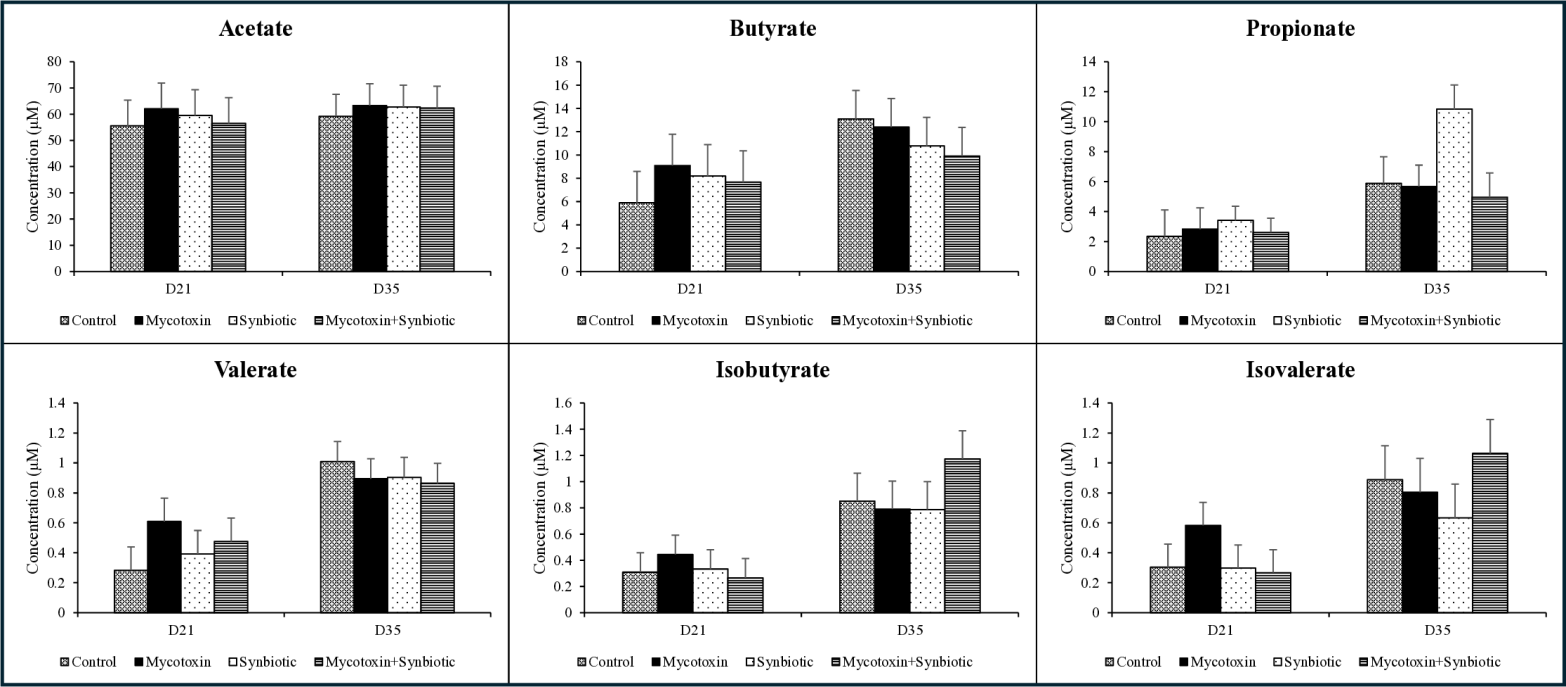
Effects of synbiotic supplementation on short-chain fatty acid concentrations of cecal contents of birds fed mycotoxin-contaminated diets on d21 and d35. The birds were supplemented with 0.05% synbiotic from d0 to d35 *(n=6)*. Mycotoxin-contaminated diets were fed from d0 to d35 in a 2×2 factorial setup. Mycotoxin- 8.5mg/kg FUM + 3.9mg/kg DON. Synbiotic- 0.05%. On d21and d35, cecal digesta was analyzed for the SCFA concentrations. Bars (± SEM) with no common superscript differ significantly (*p* < 0.05). *p*-values of d21 SCFA: 1) Acetate: mycotoxin*synbiotic = 0.63, mycotoxin = 0.86, synbiotic = 0.94; 2) Butyrate: mycotoxin*synbiotic = 0.50, mycotoxin = 0.63, synbiotic = 0.87; 3) Propionate: mycotoxin*synbiotic = 0.50, mycotoxin = 0.87, synbiotic = 0.66; 4) Valerate: mycotoxin*synbiotic = 0.48, mycotoxin = 0.21, synbiotic = 0.94; 5) Isobutyrate: mycotoxin*synbiotic = 0.50, mycotoxin = 0.83, synbiotic = 0.61; 6) Isovalerate: mycotoxin*synbiotic = 0.33, mycotoxin = 0.43, synbiotic = 0.31. *p* values of D35 SCFA: 1) Acetate: mycotoxin*synbiotic = 0.80, mycotoxin = 0.83, synbiotic = 0.88; 2) Butyrate: mycotoxin*synbiotic = 0.97, mycotoxin = 0.75, synbiotic = 0.34; 3) Propionate: mycotoxin*synbiotic = 0.09, mycotoxin = 0.07, synbiotic = 0.20; 4) Valerate: mycotoxin*synbiotic = 0.77, mycotoxin = 0.57, synbiotic = 0.61; 5) Isobutyrate: mycotoxin*synbiotic = 0.30, mycotoxin = 0.45, synbiotic = 0.46; 6) Isovalerate: mycotoxin*synbiotic = 0.26, mycotoxin = 0.45, synbiotic = 0.99.

On d21 and d35, there were no significant interactions or main effects of FUM + DON and synbiotic supplementation on acetate, butyrate, propionate, valerate, isobutyrate, and isovalerate concentrations (*p* > 0.05) (**Figure 3**). However, a trend was observed in the main effect of FUM + DON on the propionate concentrations on d35 (*p* = 0.09), where FUM + DON decreased the cecal propionate concentrations by 36.4% compared with the no mycotoxin treatment groups.

### 3.6 Correlation analysis between cecal microbial families and SCFA concentrations

**Figure 4:**
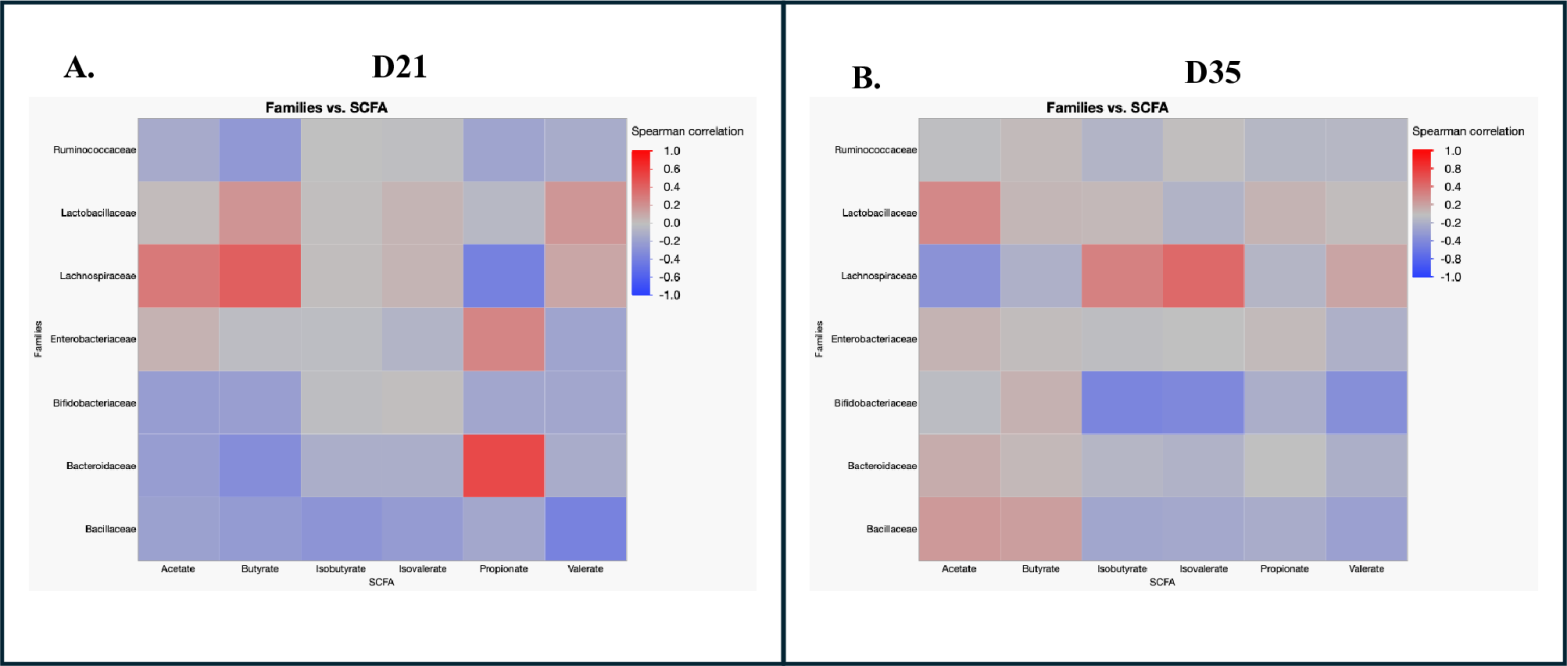
Correlation between major cecal microbial families and short-chain fatty acid (SCFA) concentrations in broiler chickens on d21 and d35. Heatmaps show Spearman’s rank correlation coefficients (ρ) between the relative abundance of dominant microbial families and cecal SCFA concentrations, including acetate, butyrate, isobutyrate, isovalerate, propionate, and valerate, on d21 **(A)** and d35 **(B)**. The intensity of color represents the strength and direction of correlations, with red indicating positive correlations and blue indicating negative correlations. *p*- values for d21 and d35 are not significant after FDR correction.

After false discovery rate (FDR) correction, no significant correlations were observed between cecal microbial families and short-chain fatty acid concentrations on days 21 and 35 (*p* > 0.05) (**Figures 4A, B****).**

### 3.7 Correlation analysis between microbial phyla and immune parameters

After FDR correction, a significant positive correlation was observed between the relative abundance of Cyanobacteria and serum DON-specific IgY concentrations (ρ = 0.6228, FDR-adjusted *p* = 0.0336) on d35 (**Supplementary Figure 1**). No significant correlations were detected between any other microbial phyla and immune parameters (*p* > 0.05).

### 3.8 Effect of synbiotic supplementation on the cecal microbiota of broiler chickens fed mycotoxin-contaminated diets on d21

**Figure 5:**
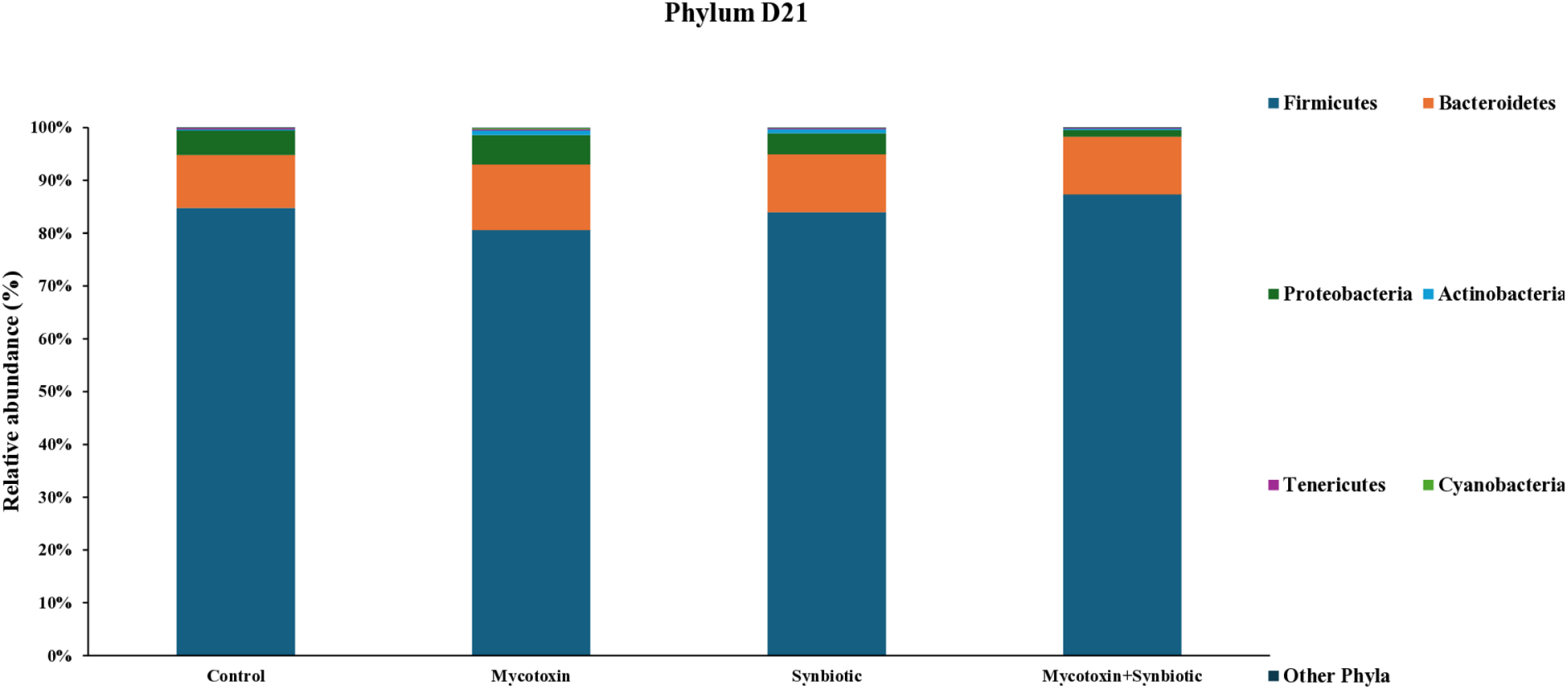
Effect of synbiotic supplementation on the relative abundance of cecal microbial composition at the phylum level in broiler chickens fed mycotoxin‑contaminated diets on d21. Birds were supplemented with 0.05% synbiotic from d0 to d35 (n = 6). Mycotoxin‑contaminated diets were fed from d0 to d35 in a 2 × 2 factorial setup. Mycotoxin treatment: 8.5 mg/kg FUM + 3.9 mg/kg DON. Synbiotic treatment: 0.05%. On d21, cecal digesta was analyzed for relative microbial abundance using 16S rRNA sequencing.

**Figure 6:**
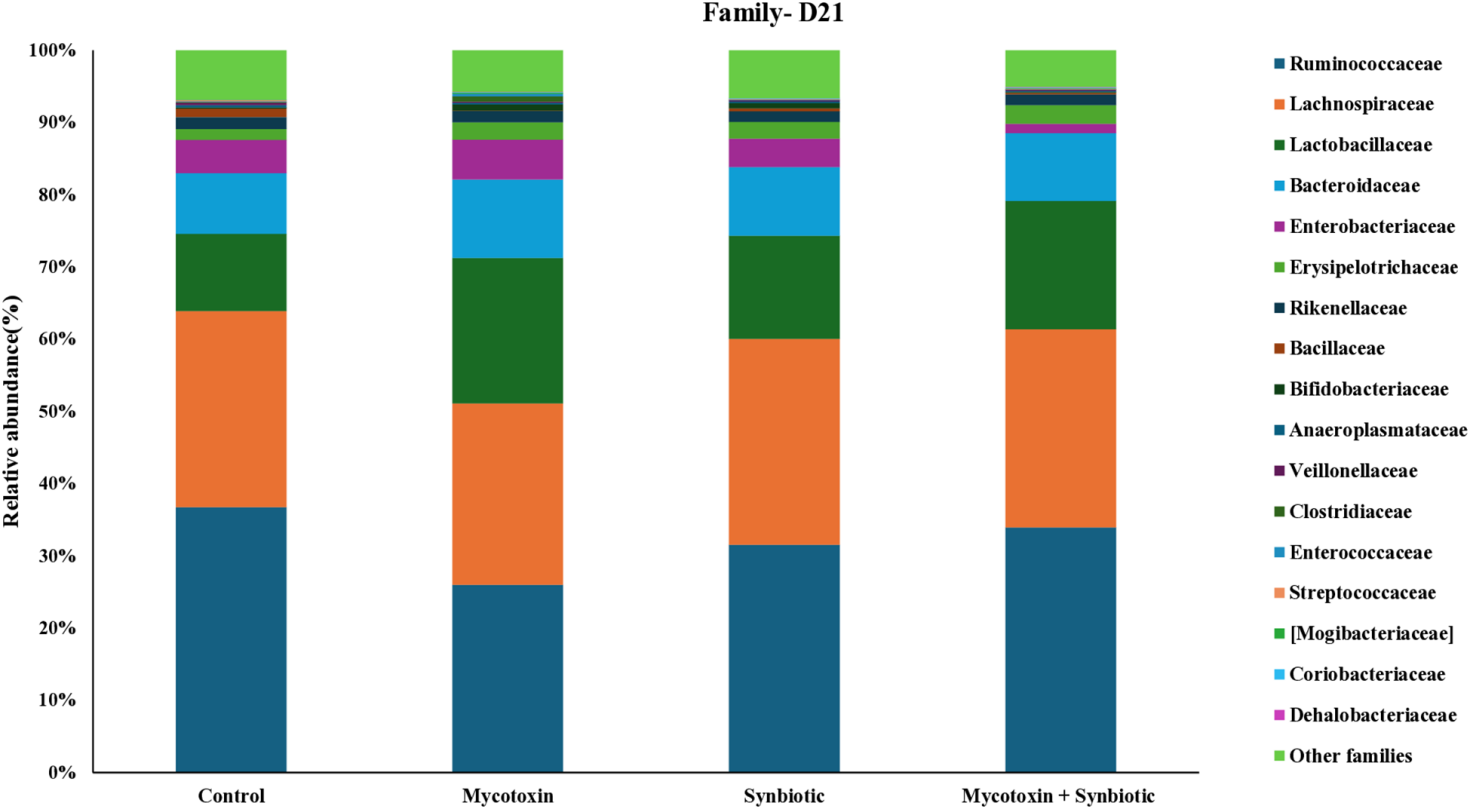
Effect of synbiotic supplementation on the relative abundance of cecal microbial composition at the family level in broiler chickens fed mycotoxin‑contaminated diets on d21. Birds were supplemented with 0.05% synbiotic from d0 to d35 (n = 6). Mycotoxin‑contaminated diets were fed from d0 to d35 in a 2 × 2 factorial setup. Mycotoxin treatment: 8.5 mg/kg FUM + 3.9 mg/kg DON. Synbiotic treatment: 0.05%. On d21, cecal digesta was analyzed for relative microbial abundance using 16S rRNA sequencing.

**Figure 7:**
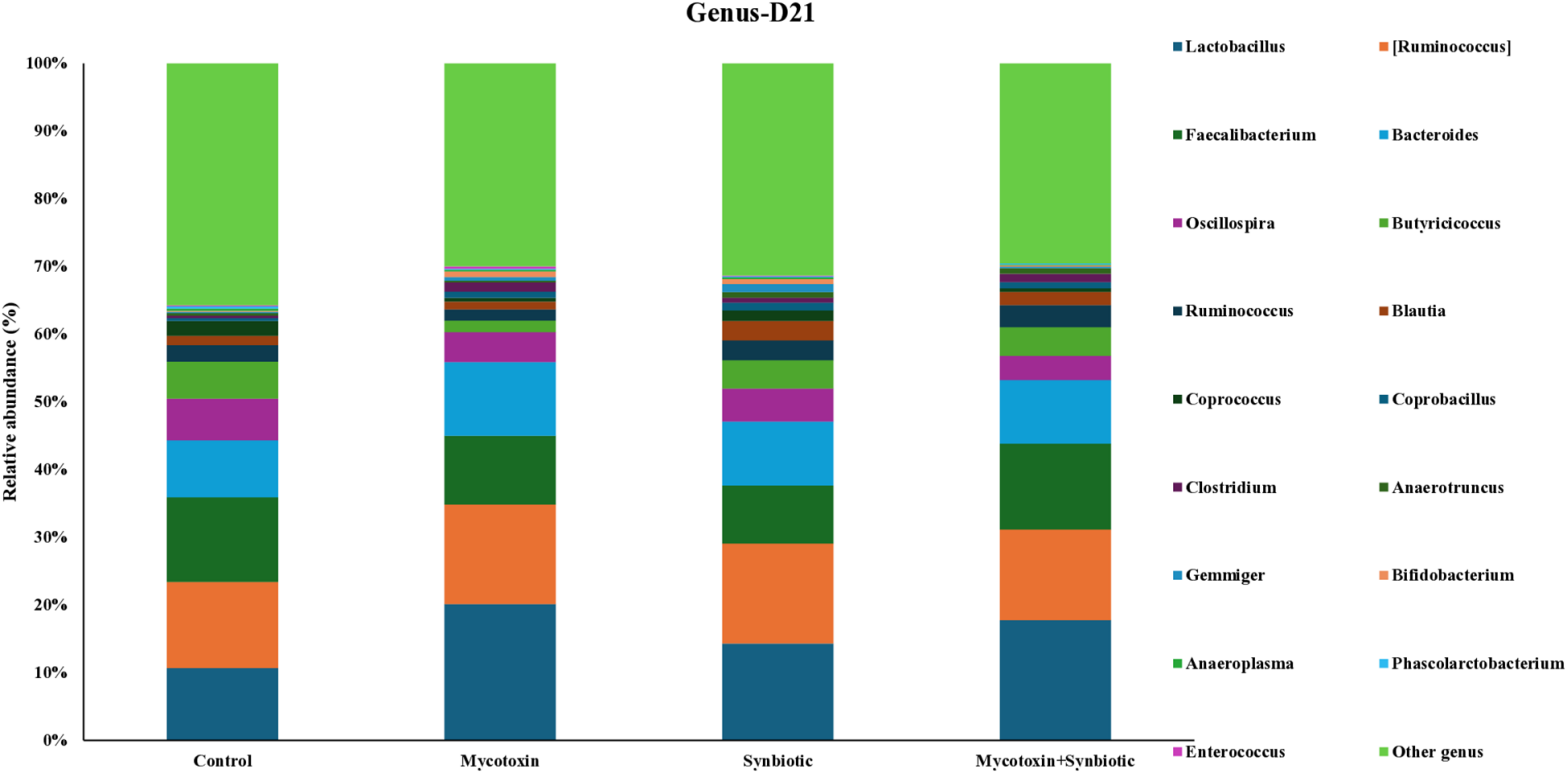
Effect of synbiotic supplementation on the relative abundance of cecal microbial composition at the genus level in broiler chickens fed mycotoxin‑contaminated diets on d21. Birds were supplemented with 0.05% synbiotic from day 0 to day 35 (n = 6). Mycotoxin‑contaminated diets were fed from day 0 to day 35 in a 2 × 2 factorial setup. Mycotoxin treatment: 8.5 mg/kg FUM + 3.9 mg/kg DON. Synbiotic treatment: 0.05%. On d21, cecal digesta was analyzed for relative microbial abundance using 16S rRNA sequencing.

On d21, six phyla were identified, with *Firmicutes* being most abundant, followed by *Bacteroidetes, Proteobacteria, and Actinobacteria* (**Figure 5**). At the family level, 24 families were detected with *Ruminococcaceae* being the most predominant, followed by Lachnospiraceae, Lactobacillaceae, and Bacteroidaceae (**Figure 6**). At the genus level, 30 genera were identified on d21, with Lactobacillus being the most abundant, followed by Ruminococcus, Faecalibacterium, and Bacteroides (**Figure 7**). FUM + DON and synbiotic supplementation did not significantly impact cecal microbiota composition at the phylum, family, and genus level on d21 (*p* > 0.1).

**Figure 8:**
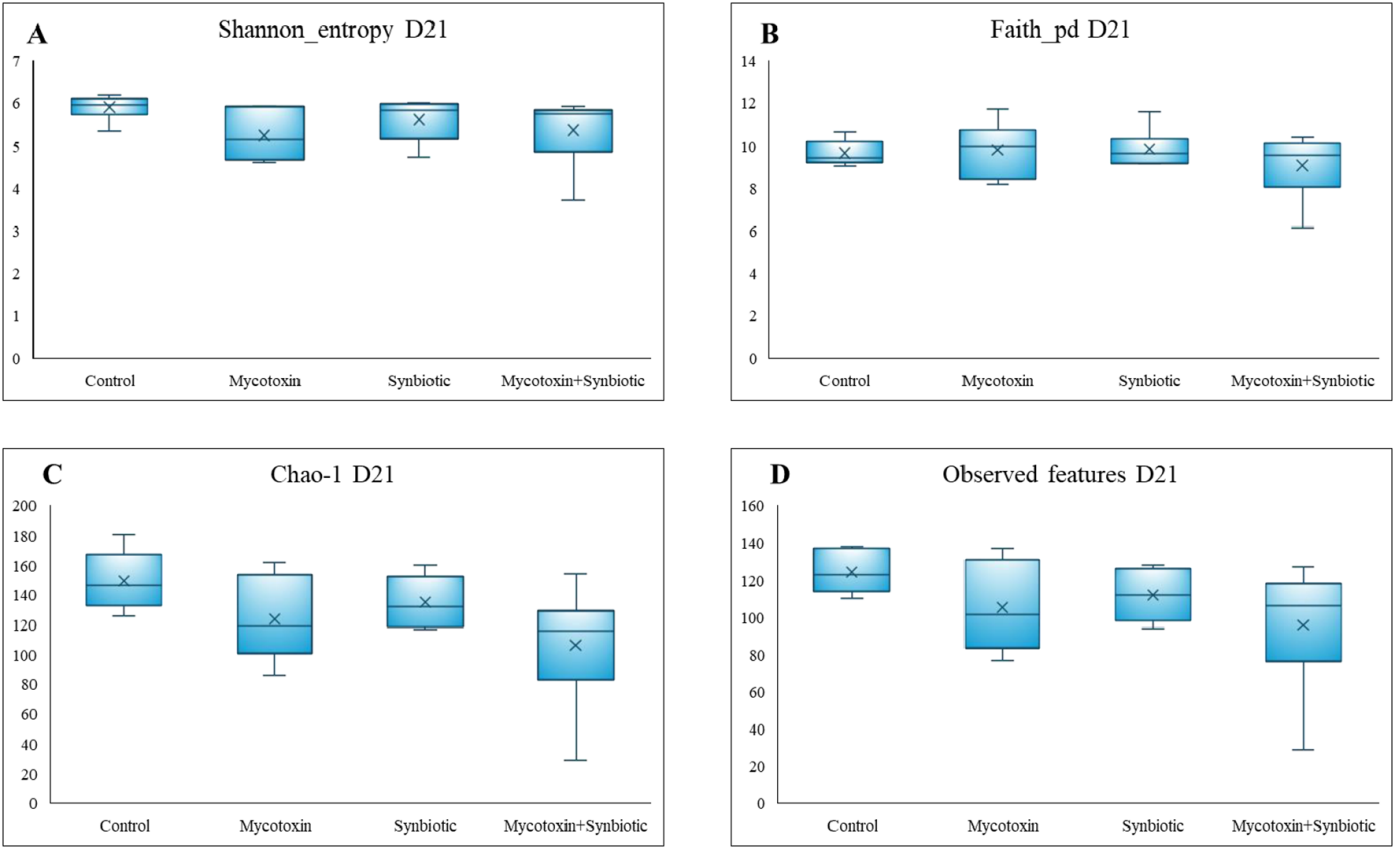
Effect of synbiotic supplementation on alpha diversity indices of broiler chickens fed mycotoxin‑contaminated diets on d21. **(A)** Shannon diversity index (p > 0.05), **(B)** Faith’s phylogenetic diversity index (p > 0.05), **(C)** Chao‑1 abundance index (p = 0.09), and **(D)** number of observed features (p > 0.05). Birds were supplemented with 0.05% synbiotic from day 0 to day 35. Mycotoxin‑contaminated diets were fed from day 0 to day 35 in a 2 × 2 factorial design. Mycotoxin treatment: 8.5 mg/kg FUM + 3.9 mg/kg DON. Synbiotic treatment: 0.05%. On d21, cecal digesta was analyzed for relative microbial abundance using 16S rRNA sequencing (n = 6).

On d21, a trend was observed for the Chao-1 index (p = 0.09), which decreased in the FUM + DON and synbiotic + FUM + DON groups compared to controls (**Figure 8**). No significant differences were observed for Shannon diversity, Faith’s phylogenetic diversity, or observed features (*p* > 0.1).

### 3.9 Effect of synbiotic supplementation on the cecal microbiota of broiler chickens fed mycotoxin-contaminated diets on d35

**Figure 9:**
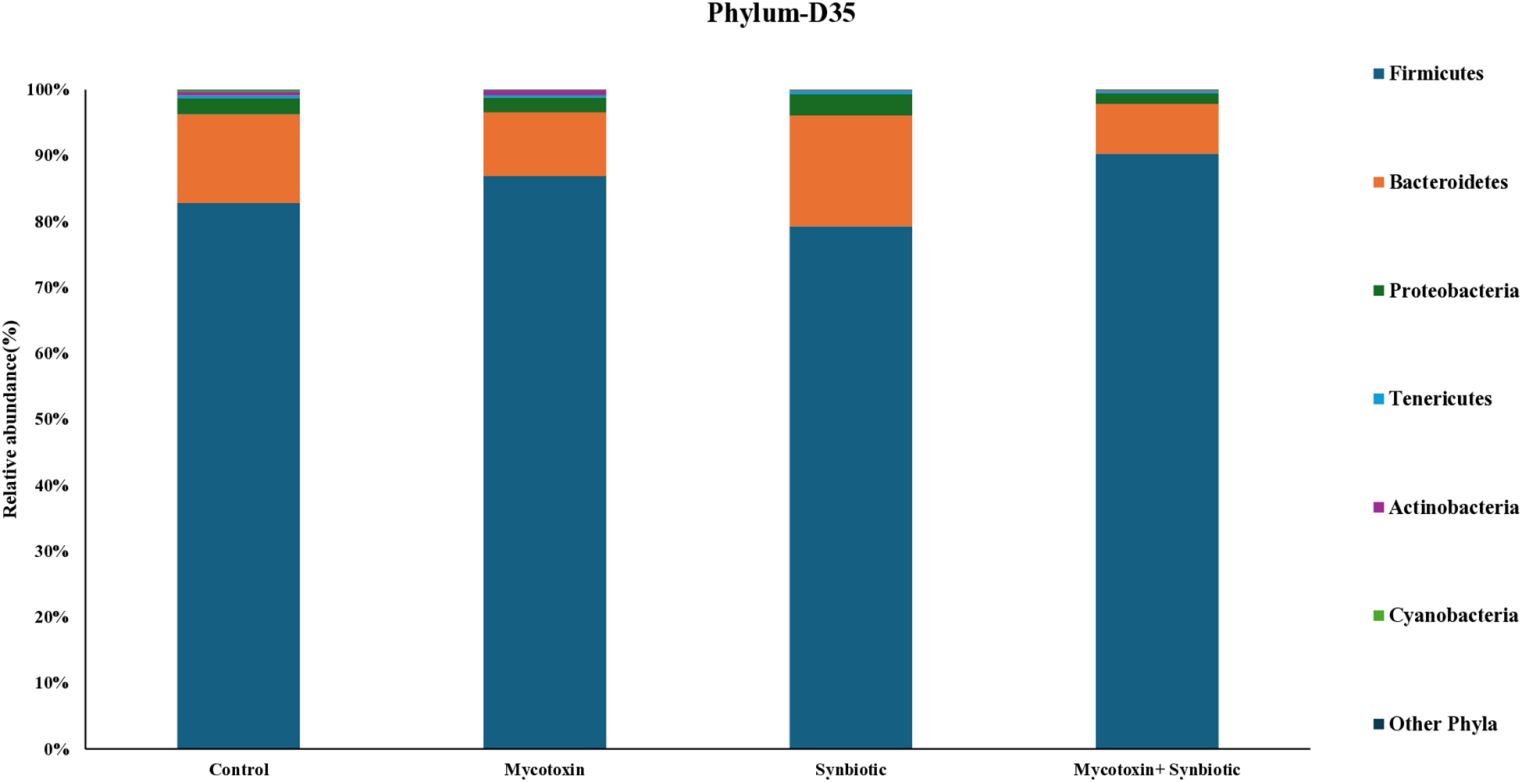
Effect of synbiotic supplementation on the relative abundance of cecal microbial composition at the phylum level in broiler chickens fed mycotoxin‑contaminated diets on d35. Birds were supplemented with 0.05% synbiotic from day 0 to day 35 (n = 6). Mycotoxin‑contaminated diets were fed from day 0 to day 35 in a 2 × 2 factorial setup. Mycotoxin treatment: 8.5 mg/kg FUM + 3.9 mg/kg DON. Synbiotic treatment: 0.05%. On d35, cecal digesta was analyzed for relative microbiome abundance using 16S rRNA sequencing.

**Figure 10:**
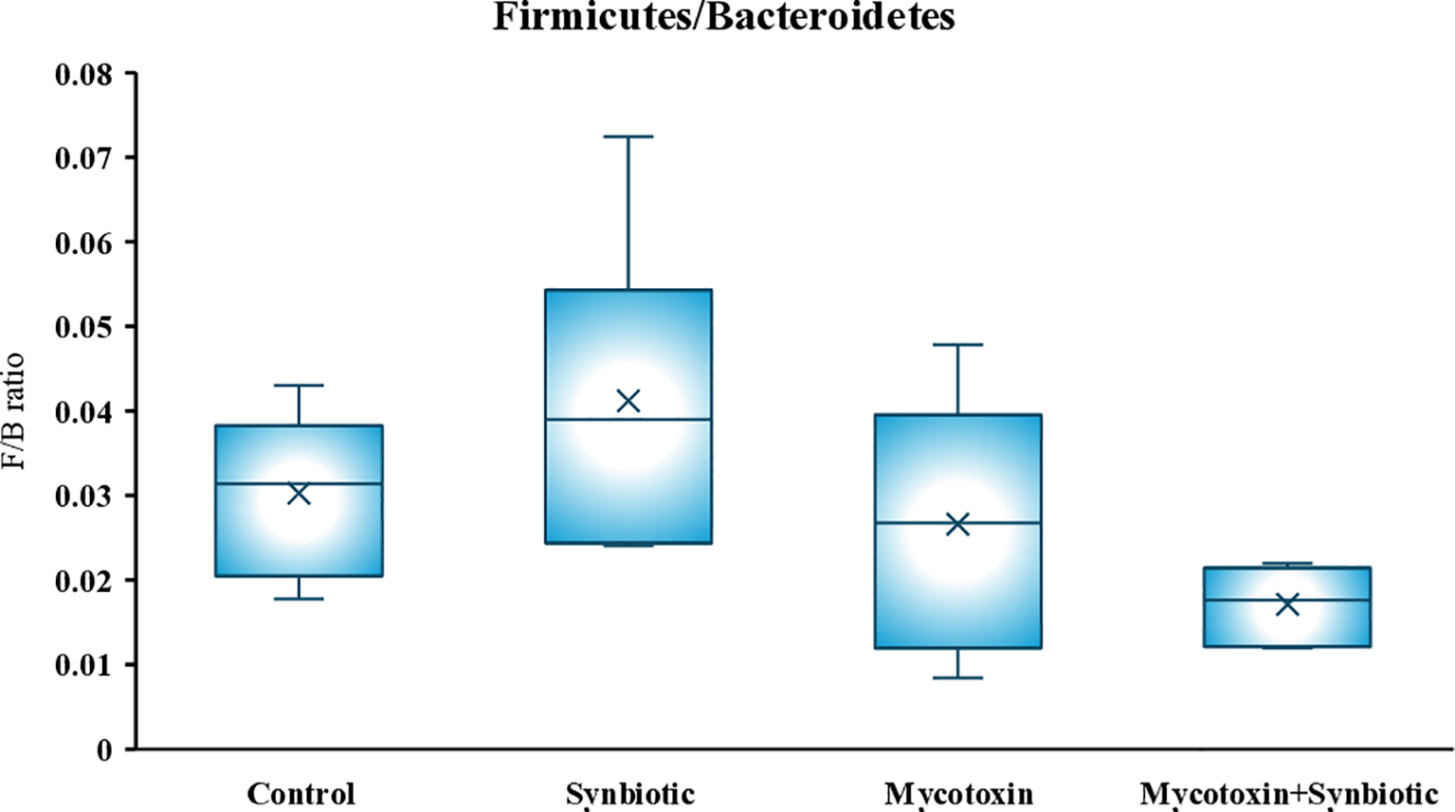
Effect of synbiotic supplementation on relative abundance of cecal microbial composition at the phylum level of broiler chickens fed mycotoxin-contaminated diets on d35. The birds were supplemented with 0.05% synbiotic from d0 to d35 *(n=6)*. Mycotoxin-contaminated diets were fed from d0 to d35 in a 2×2 factorial setup. Mycotoxin- 8.5mg/kg FUM + 3.9mg/kg DON. Synbiotic- 0.05%. On d35, cecal digesta was analyzed for the relative abundance of microbiome using 16S rRNA sequencing. Phyla highlighted in red boxes have significant differences (*p* < 0.1).

On d35, six phyla were identified, similar to d21. Significant differences were observed in the relative abundance of *Firmicutes, Bacteroidetes, and Proteobacteria* among treatment groups (*p* < 0.1) (**Figure 9**). There were significant differences in the relative abundance of *Firmicutes, Bacteroidetes, and Proteobacteria* among the treatment groups (*p* < 0.1). Firmicutes significantly increased by 4% in the FUM + DON group and by 8% in the synbiotic + FUM + DON group compared to the control group (*p* < 0.1). In contrast, FUM + DON decreased the relative abundance of Bacteroidetes by 3.7% and by 5.8% in the synbiotic + FUM + DON group compared to the control group (*p* < 0.1). These shifts resulted in a significant increase in the Firmicutes-to-Bacteroidetes (F/B) ratio among treatments (*p* < 0.1), with birds in the FUM + DON and synbiotic + FUM + DON group showing markedly higher F/B ratios compared with the control, while synbiotic supplementation alone reduced the F/B ratio (**Figure 10**). The relative abundance of Proteobacteria decreased by 0.24% in the FUM + DON group and decreased by 0.9% in the synbiotic + FUM + DON group compared to the control group (*p* < 0.1).

**Figure 11:**
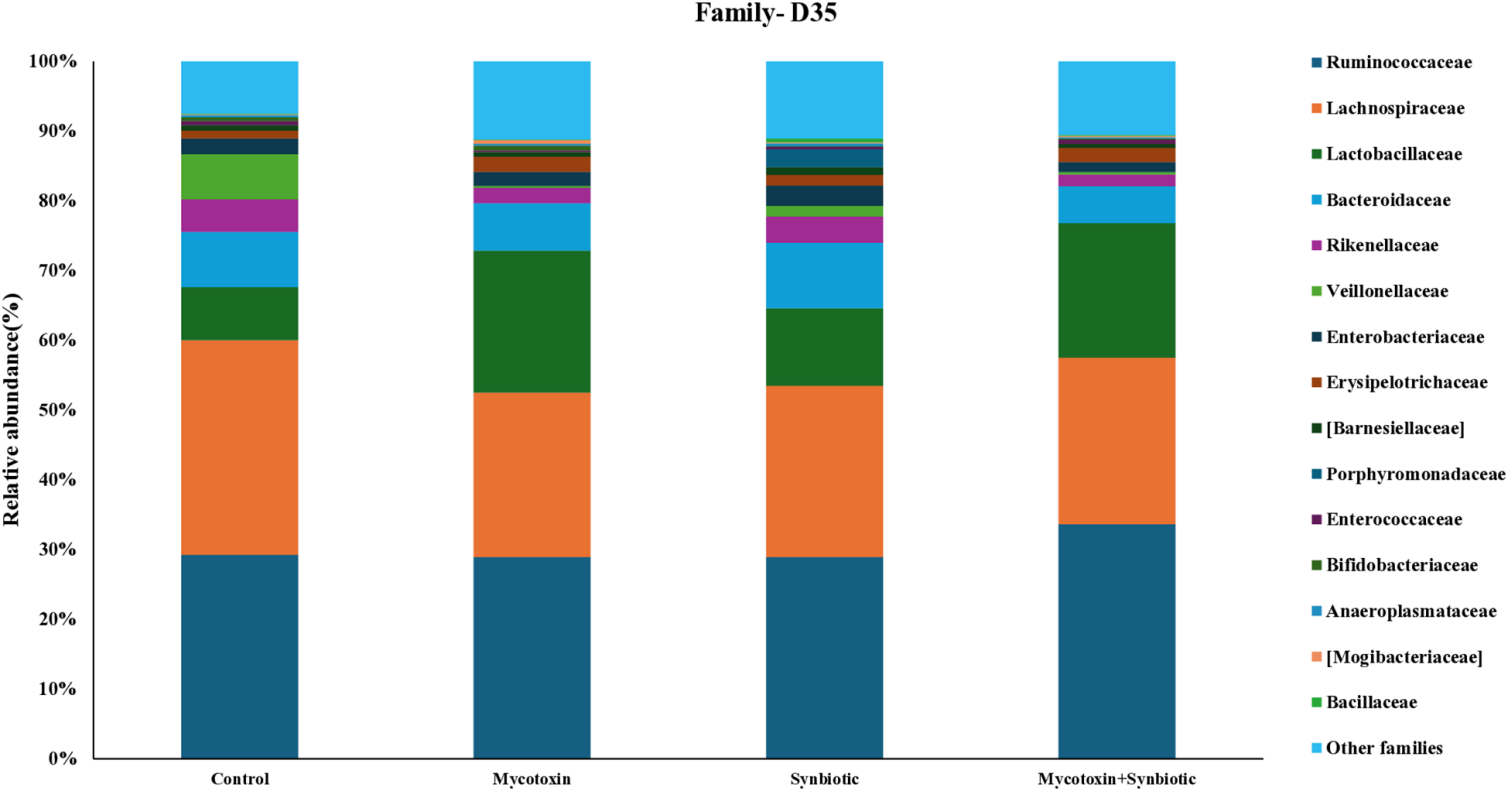
Effect of synbiotic supplementation on the relative abundance of cecal microbial composition at the family level in broiler chickens fed mycotoxin‑contaminated diets on d35. Birds were supplemented with 0.05% synbiotic from day 0 to day 35 (n = 6). Mycotoxin‑contaminated diets were provided from day 0 to day 35 in a 2 × 2 factorial design. Mycotoxin treatment: 8.5 mg/kg FUM + 3.9 mg/kg DON. Synbiotic treatment: 0.05%. On d35, cecal digesta was analyzed for relative microbial abundance using 16S rRNA sequencing.

**Figure 12:**
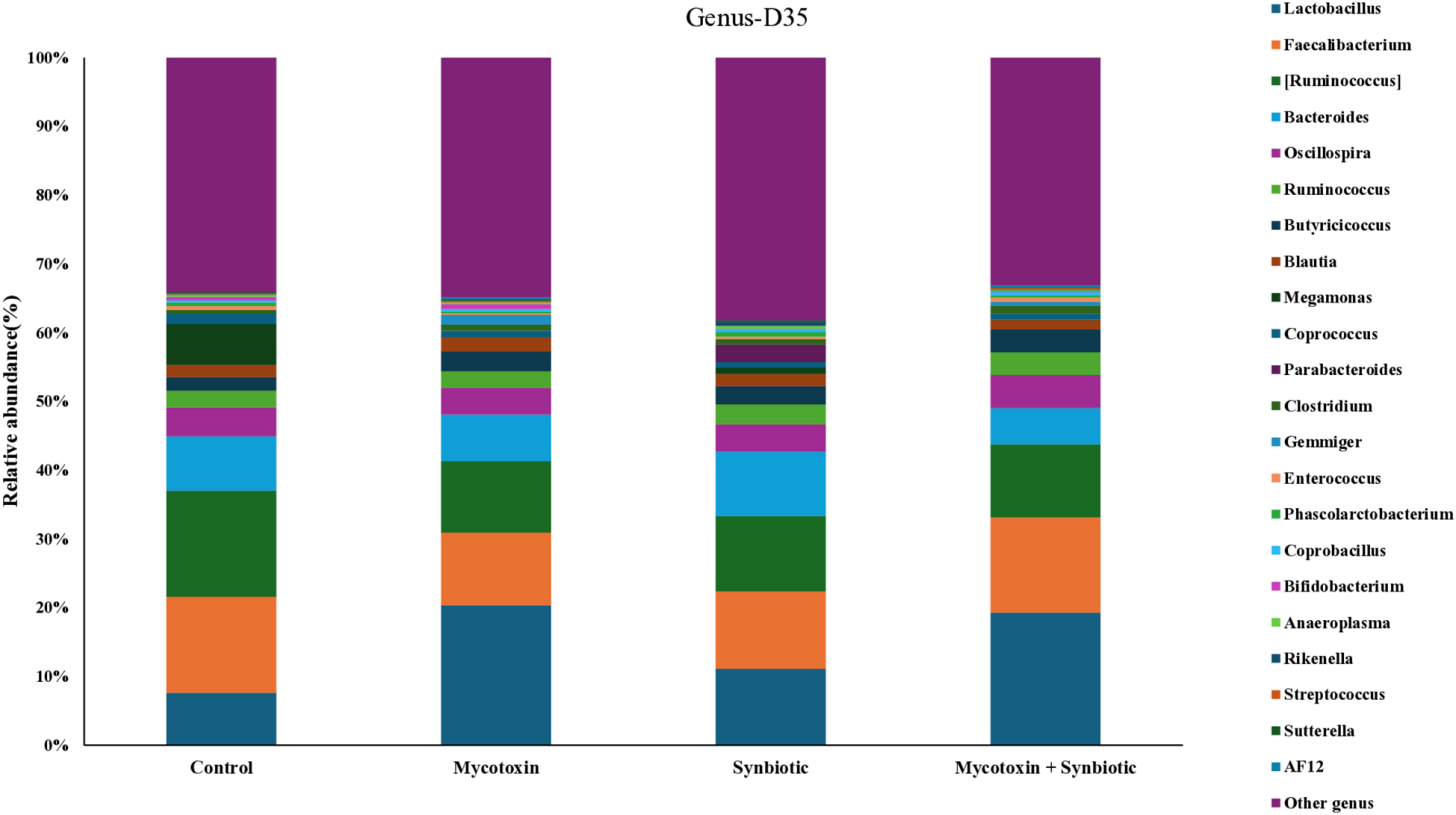
Effect of synbiotic supplementation on the relative abundance of cecal microbial composition at the genus level in broiler chickens fed mycotoxin‑contaminated diets on d35. Birds were supplemented with 0.05% synbiotic from day 0 to day 35 (n = 6). Mycotoxin‑contaminated diets were provided from day 0 to day 35 in a 2 × 2 factorial design. Mycotoxin treatment: 8.5 mg/kg FUM + 3.9 mg/kg DON. On d35, cecal digesta was analyzed for relative microbial abundance using 16S rRNA sequencing.

A total of 30 families were identified at the family level. On d35, the most predominant group in the ceca was Ruminococcaceae, followed by Lachnospiraceae, Lactobacillaceae, and Bacteroidaceae, in all the treatment groups (**Figure 11**). Among the treatment groups, a total of 39 genera were identified, and the most abundant genera on d35 were *Lactobacillus*, followed by *Faecalibacterium*, *Ruminococcus*, and *Bacteroides* (**Figure 12**). *Faecalibacterium* relative abundance was the most predominant over the *Ruminococcus* on d35 compared to d21 cecal microbiota at the genus level. FUM + DON and synbiotic supplementation did not significantly alter the cecal microbiota on d21 at the family and genus level.

**Figure 13:**
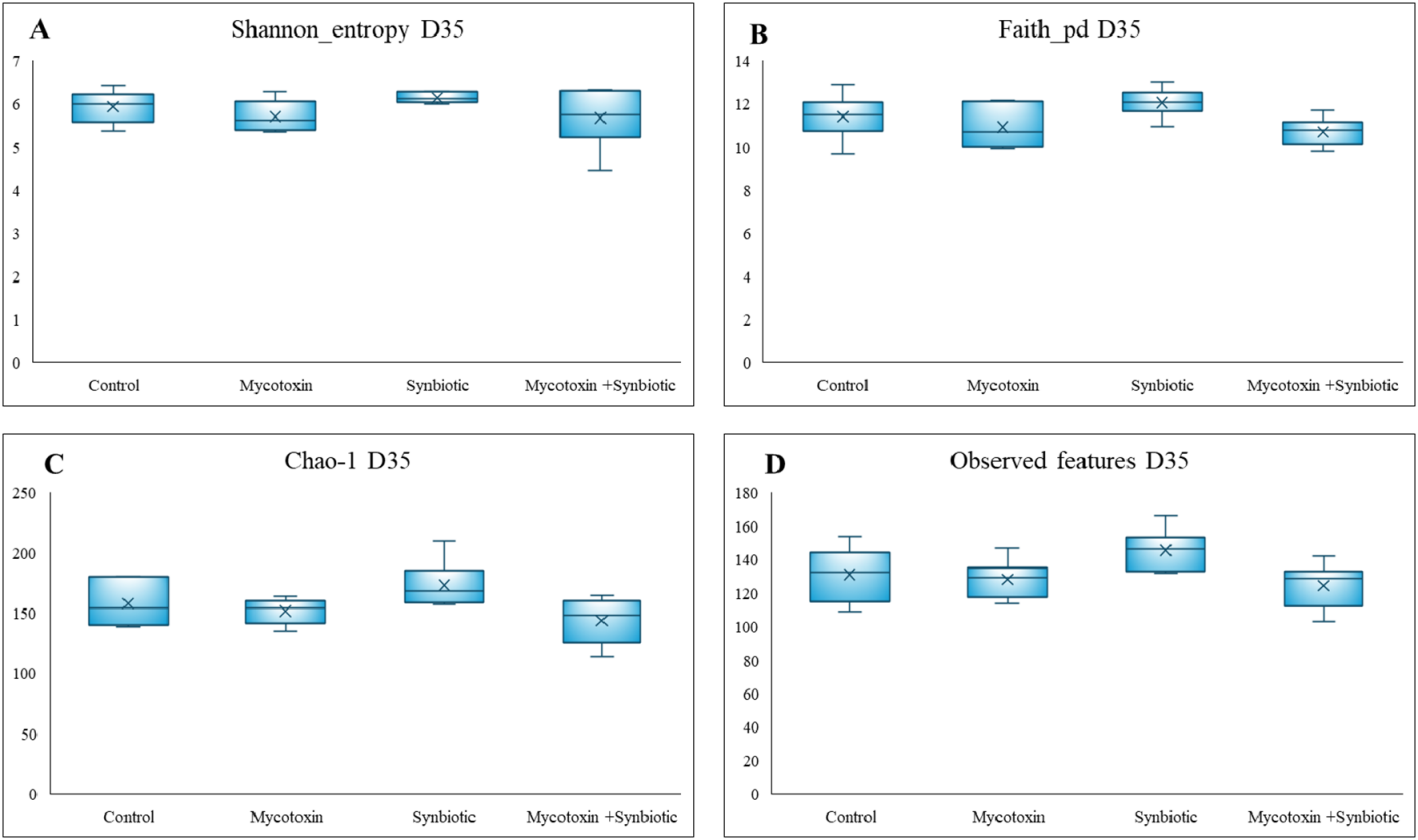
Effect of synbiotic supplementation on alpha diversity indices of broiler chickens fed mycotoxin-contaminated diets on d35. **(A)**- Shannon diversity (p > 0.05), **(B)**- Faith phylogenetic diversity index (p = 0.06), **(C)**- Chao-1 abundance index (p > 0.05), **(D)**- Number of observed features (p = 0.056). Birds were supplemented with 0.05% synbiotic from d0 to d35. Mycotoxin-contaminated diets were fed from d0 to d35 in a 2 x 2 factorial setup. Mycotoxin- 8.5 mg/kg FUM + 3.9 mg/kg DON. Synbiotic- 0.05%. On d35, cecal digesta was analyzed for the relative abundance of microbiome using 16S rRNA sequencing (n=6).

**Figure 14:**
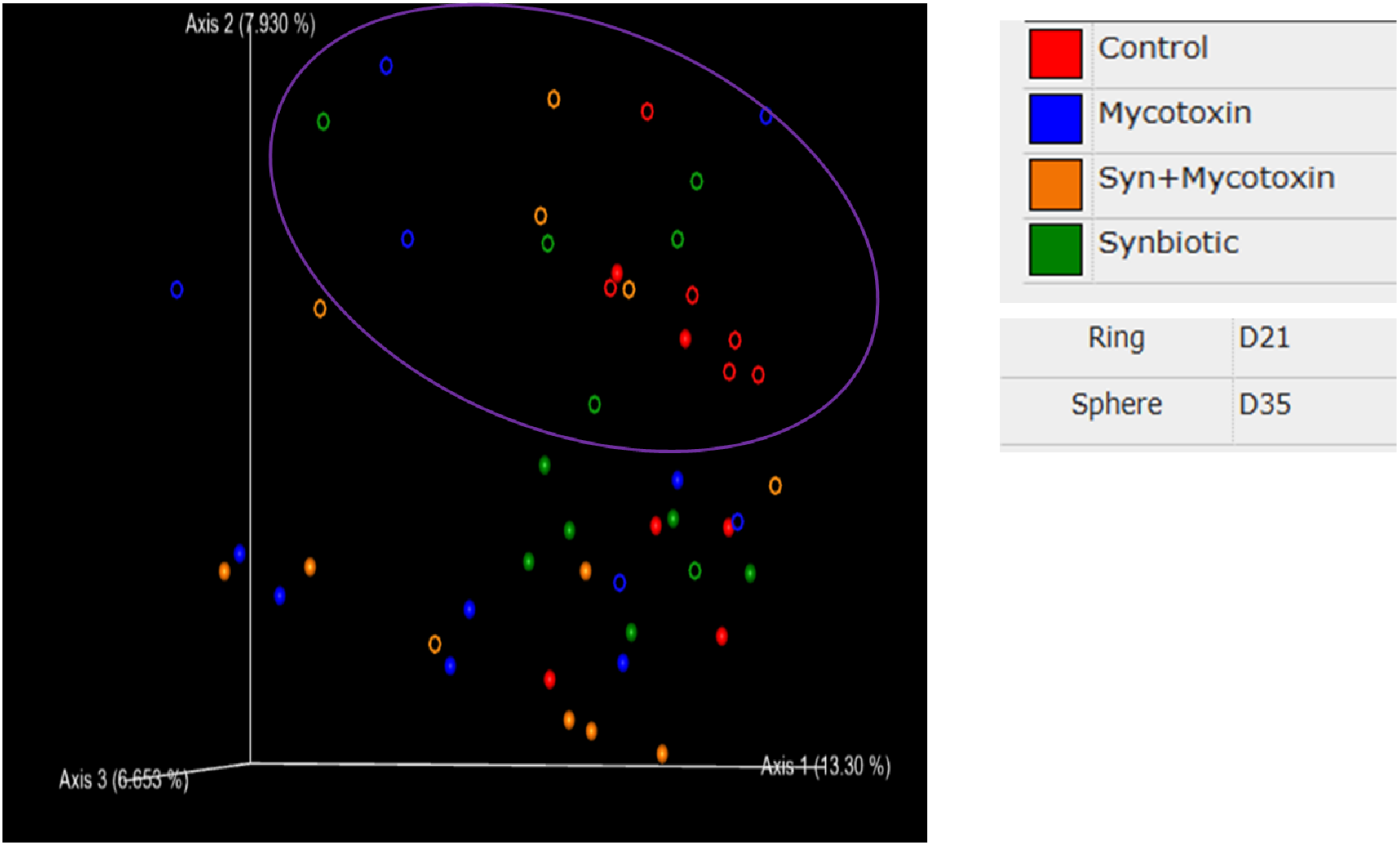
Effect of synbiotic supplementation on beta-diversity based on Jaccard distances of cecal microbiota on d21 and d35. Birds were supplemented with 0.05% synbiotic from d0 to d35 (n=6). Mycotoxin-contaminated diets were fed from d0 to d35 in a 2 x 2 factorial setup. Mycotoxin- 8.5 mg/kg FUM + 3.9 mg/kg DON. Synbiotic- 0.05%. On d21 and d35, cecal digesta were analyzed for the relative abundance of microbiome using 16S rRNA sequencing.

On d35, Synbiotic supplementation had a significant effect on the number of observed features and the Faith phylogenetic diversity index. However, there was a trend in the number of observed features (*p* = 0.05) and Faith phylogenetic diversity index (*p* = 0.06) among the treatment groups (**Figure 13**), both of which decreased in the FUM + DON group and the synbiotic + FUM + DON group compared to controls. No differences were observed for Shannon diversity and Chao-1 indexes (*p* > 0.1). No differences were observed for Shannon diversity, beta diversity (**Figure 14**) and Chao-1 indexes (*p* > 0.1).

### 3.10 Effect of synbiotic supplementation on predicted microbial metabolic pathways

**Figure 15:**
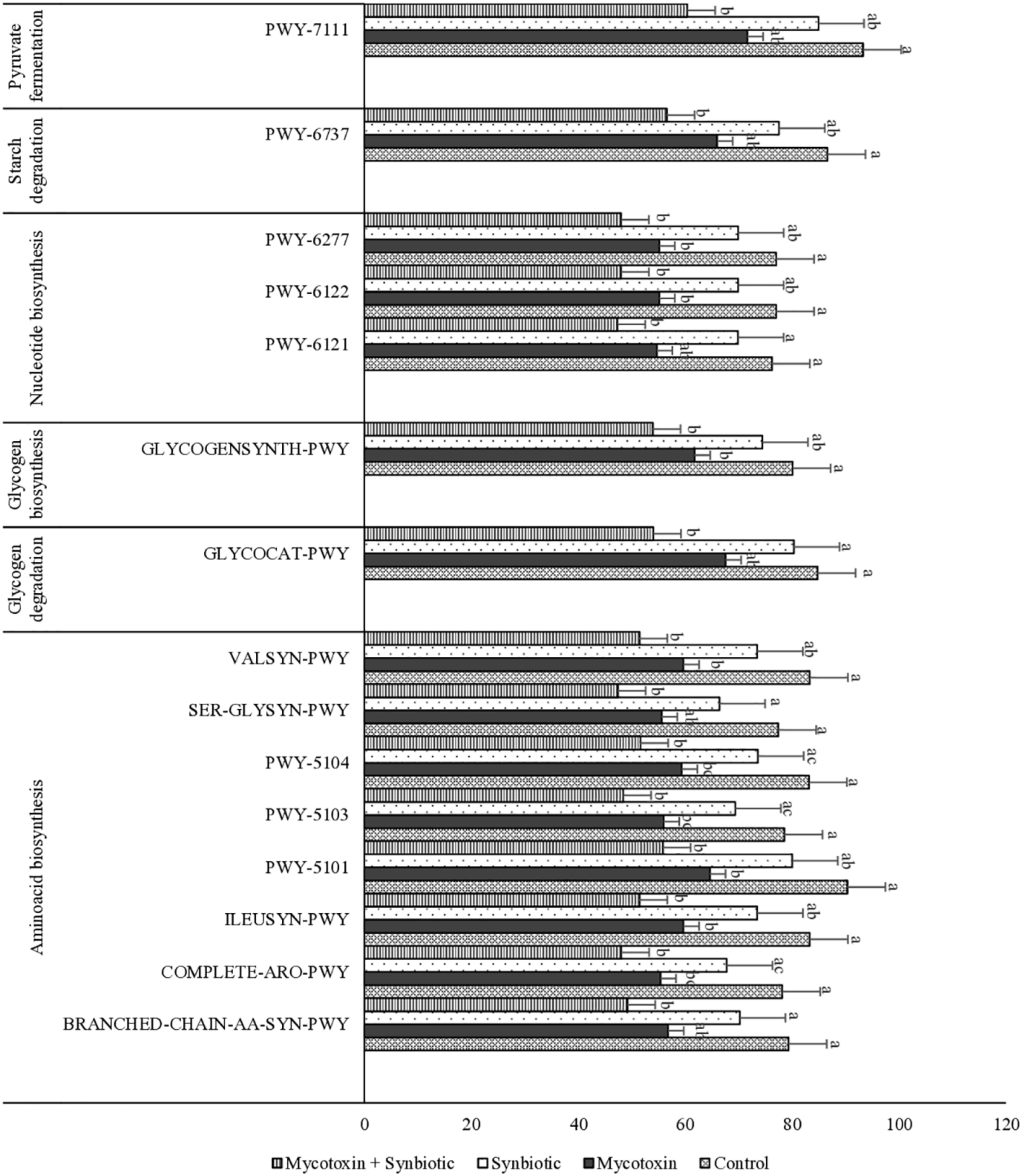
Predicted microbial metabolic pathways involved in amino acid biosynthesis, carbohydrate metabolism, nucleotide biosynthesis, starch degradation, and pyruvate fermentation in broiler chickens exposed to fumonisins and deoxynivalenol with or without synbiotic supplementation on day 35. Pathways were compared among treatment groups using the Kruskal–Wallis test, and p-values were adjusted for multiple comparisons using the Benjamini–Hochberg false discovery rate (FDR) method. Only pathways with significant differences among treatments (p < 0.05) are shown.

On d35, significant differences were observed in amino acid, carbohydrate, and nucleotide metabolism pathways among treatment groups (*p* < 0.05) (**Figure 15**). Multiple amino acid biosynthesis pathways, including branched-chain amino acid synthesis (BRANCHED-CHAIN-AA-SYN-PWY), aromatic amino acid synthesis (COMPLETE-ARO-PWY), and valine biosynthesis (VALSYN-PWY), were significantly downregulated in birds exposed to mycotoxin compared with control. Similarly, pathways involved in glycogen degradation (GLYCOCAT-PWY), glycogen biosynthesis (GLYCOGENSYNTH-PWY), starch degradation (PWY-6737), and pyruvate fermentation (PWY-7111), and nucleotide biosynthesis pathways (PWY-6121, PWY-6122, PWY-6277) were significantly downregulated in the mycotoxin group compared to controls. Synbiotic supplementation had no significant differences in carbohydrate and fermentation related pathways compared to controls (*p* > 0.5).

## 4 Discussion

This study evaluated the impact of 0.05% synbiotic supplementation on humoral immune response, SCFAs profile, and cecal microbiota composition in broilers exposed to subclinical doses of FUM and DON. Although the combined FUM+DON challenge did not induce visible clinical symptoms, measurable alterations in microbial structure, predicted metabolic pathways, and trends in mucosal antibody responses were observed, indicating subtle but biologically relevant intestinal disturbances. In our previous report from this same experimental trial, synbiotic supplementation at 0.05% reversed the mycotoxin-induced decrease in production performance and immune response when chickens were exposed to 8.7 mg FUM + 3.9 mg DON per kg diet (Dasireddy et al., 2025). In the current study, our goal was to identify whether the altered production performance and immune response were associated with the altered relative abundance of the cecal microbiome. Our findings indicate that synbiotic supplementation counteracted the negative impact of FUM + DON exposure by improving DON-specific IgA responses and modulating gut microbial composition, although it did not fully restore microbial diversity or serum IgY levels.

It is important to note that low background levels of FUM, DON, and ZEA were also detected in the control and synbiotic diets. This low−level contamination is common and largely unavoidable in poultry nutrition because cereal−based ingredients naturally contain trace mycotoxins even when high−quality sources are used. Therefore, the control and synbiotic diets should be interpreted as “lowest practical exposure” rather than completely mycotoxin−free baselines. The treatment diets provided a clearly higher and controlled exposure to FUM + DON, allowing assessment of the effects of increased mycotoxin challenge under realistic production conditions.

Secretory immunoglobulins play a critical role in mucosal immunity by defending against pathogens and maintaining gut homeostasis (Ren et al., 2019). In the present study, no significant interaction effects were observed in FUM-specific IgA among treatment groups. However, FUM-specific IgA concentrations were decreased in mycotoxin-contaminated treatment groups. This finding is consistent with previous studies in piglets exposed to FB1 at 1mg/kg body weight for 10 days (Devriendt et al., 2009) and chickens exposed to FUM (1–33 mg/kg) + DON (0.5–3 mg/kg) for 35 days have shown a decreased total IgA concentration (Shanmugasundaram et al., 2025). Even in the absence of statistically significant differences, the observed numerical reduction in toxin-specific IgA may reflect early immunomodulatory effects at the mucosal surface (Rousseaux et al., 2023). In the current study, synbiotic supplementation showed a tendency to increase DON-specific IgA concentrations in the FUM + DON-exposed treatment group, suggesting a selective enhancement of mucosal immunity. However, in the current study, synbiotic supplementation did not reverse the decreased serum FUM and the DON-specific decreased IgY concentration in the FUM + DON treatment groups. Similar results were observed when laying hens were exposed to 12 mg DON + 0.5 mg 15-acetyl DON + 0.6 mg ZEA per kg of diet for 12 weeks; mycotoxin detoxifier supplementation failed to improve systemic antibody response (Chowdhury et al., 2005). Although serum IgY concentrations were not altered, the localized modulation of IgA suggests that synbiotics primarily influence mucosal rather than systemic humoral responses, which is consistent with the site of mycotoxin exposure.

In the current study, on both d21 and d35, cecal SCFA concentrations were largely unaffected by treatment; however, FUM + DON tended to decrease propionate concentration on d35. Propionate is primarily produced by Bacteroidetes via the succinate pathway (Döring and Basen, 2024), and its reduction corresponded with the decreased relative abundance of *Bacteroides, Veillonella, and Prevotella* in mycotoxin-exposed birds (Shanmugasundaram et al., 2023); (Döring and Basen, 2024). Propionate plays important roles in maintaining gut integrity and regulating immune response (Rychlik, 2020); (Liu et al., 2021). Therefore, even a trend toward reduced propionate production may indicate impaired microbial fermentation efficiency and compromised gut function under mycotoxin exposure. Correlation analysis between microbial families and SCFA concentrations did not show any statistically significant associations after FDR correction on either d21 or d35. In contrast to SCFA correlations, a significant positive association was identified between Cyanobacteria and serum DON-specific IgY concentrations. This finding suggests that specific microbial taxa may contribute to the modulation of systemic humoral responses under DON exposure, potentially through indirect mechanisms involving microbial metabolites. The absence of direct associations between SCFAs and immune parameters indicates that immune modulation is likely mediated through complex host–microbiota interactions rather than individual metabolites (Levy et al., 2016; Ruff et al., 2020). Interestingly, propionate levels were higher in the synbiotic-supplemented group, suggesting that the synbiotic had a targeted metabolic effect on gut microbes (Kircher et al., 2022). Such a selective increase of propionate suggests a metabolic shift toward carbohydrate fermentation rather than mycotoxin-induced protein fermentation.

PICRUSt2 based functional pathway analysis demonstrated downregulation of amino acid, carbohydrate, nucleotide synthesis and pyruvate fermentation pathways in the FUM + DON exposure group. Reduced amino acid biosynthesis pathways reflect impaired microbial anabolic capacity, potentially influencing host nutrient availability. Previous work has demonstrated that mycotoxins can disrupt amino acid metabolism and nutrient digestibility in broilers (Shanmugasundaram et al., 2025). Similarly, downregulation of carbohydrate degradation and pyruvate fermentation pathways suggests altered microbial energy metabolism (Lin et al., 2017). Synbiotic supplementation did not significantly restore all predicted pathways but appeared to maintain carbohydrate and puruvate fermentation-related pathways closer to control levels, suggesting that synbiotics likely exert protective effects through competitive exclusion, increasing beneficial taxa, which selectively stimulate commensal bacteria growth.

Mycotoxin exposure is known to disrupt gut microbial homeostasis and predispose birds to enteric infections (Antonissen et al., 2015a; Shanmugasundaram et al., 2023; Yu et al., 2022). In this study, FUM + DON decreased species richness (Chao-1 index) on d21 and Faith’s phylogenetic diversity index on d35, indicating that combined FUM + DON damaged the intestinal epithelial lining, creating a hostile environment within the gut, potentially increasing susceptibility to bacterial diseases such as necrotic enteritis and salmonellosis (Antonissen et al., 2015a; Liu J. et al., 2023; Shanmugasundaram et al., 2022); (Antonissen et al., 2015b; Shanmugasundaram et al., 2023). On the other hand, the decreased species richness in the synbiotic-supplemented groups may reflect selective activation of specific taxa rather than broad microbial expansion (Śliżewska et al., 2020). In general, higher microbial diversity is associated with better health status in poultry; however, the synbiotic bacterial strains likely failed to promote microbial diversity under conditions of altered luminal pH, which limit commensal bacteria colonization (Sicard et al., 2017). As a result, reductions in key taxa such as *Ruminococcus* and *Bacteroides* negatively influence the overall microbial diversity and their function within the chicken gut (Tudela et al., 2021).

In the current study, on d35, the predominant phylum, *Firmicutes*, relative abundance increased by 1.1-fold, while *Bacteroidetes* abundance decreased by 1.4-fold in the FUM + DON treatment group compared with controls. Although *Firmicutes* include many beneficial butyrate-producing taxa, an increased *Firmicutes*-to-*Bacteroidetes* ratio has also been associated with inflammatory dysbiosis, which may contribute to impaired tight junction integrity and increased intestinal permeability in birds exposed to FUM + DON (Hakansson and Molin, 2011). Members of the genus *Bacteroides* are opportunistic pathogens and are part of the normal gut microbiota (Patrick, 2002) play an important role in complex carbohydrate and protein metabolism. However, excessive protein fermentation by-products can potentially enhance gut inflammation and impair nutrient absorption (Fultz et al., 2021). The observed shift in a higher F/B ratio in the FUM + DON group is aligned with the previous study when chickens were exposed to 3 mg FUM + 4 mg DON per kg diet for 21 days, resulting in intestinal dysbiosis (Shanmugasundaram et al., 2023). Interestingly, synbiotic supplementation in the FUM + DON group further increased the *Firmicutes* by 1.1-fold and decreased *Bacteroidetes* by 1.1-fold relative to controls. This suggests that the synbiotic may alter microbial community dynamics during toxin exposure, which may help maintain intestinal homeostasis despite these changes in taxonomic composition.

In the present study, the relative abundance of Proteobacteria was decreased by 1.1-fold in the FUM + DON group, but the addition of synbiotics to the diet decreased it further by 0.5-fold compared to the control group. Proteobacteria are a significant component of the chicken gut microbiota and often comprise opportunistic pathogens like *E. coli, Salmonella*, and *Campylobacter* (Rychlik, 2020). A similar decrease in the relative abundance of proteobacteria was observed in broilers exposed to 3 mg FUM + 4 mg DON per kg diet (Shanmugasundaram et al., 2023). The decrease in proteobacteria by synbiotic supplementation suggests the shift toward beneficial microbes may help prevent infections caused by opportunistic pathogens.

In the present study, synbiotic supplementation did not increase the overall abundance of Actinobacteria. This is most likely due to the underestimation of Actinobacteria in 16S rRNA sequencing caused by their lower gene copy number compared to other phyla (Candela et al., 2004). *Mogibacteriaceae*, a family within Actinobacteria, showed a numerical increase in relative abundance in the FUM + DON group but decreased in the synbiotic-supplemented group, although its role in mycotoxin detoxification remains unclear. Interestingly, FUM + DON exposure decreased *Bacillaceae* relative abundance by 1.7-fold on both d21 and d35, whereas synbiotic supplementation increased *Bacillaceae* relative abundance by 1.03-fold in FUM + DON-treated groups. This is noteworthy because *Bacillus* species exert antifungal activity and can degrade mycotoxins into less toxic metabolites (Veras et al., 2023). Several species of *Bacillus* are known to have a degradation effect on mycotoxins like aflatoxin, zearalenone, ochratoxin, and DON (Liu X. et al., 2023; Shu et al., 2018; Wang et al., 2020). *Bacillus* sp. LS100 supplementation in swine mitigates the adverse effects of DON by degrading DON into less toxic metabolites (Li et al., 2011). On d21 and d 35, *Veillonellaceae*, major propionate producers (Xiao et al., 2022), decreased in the FUM + DON group, correlating with reduced propionate concentrations. On both d21 and d35, *Enterobacteriaceae*, members of Proteobacteria, numerically decreased in the FUM + DON group supplemented with synbiotics compared to the control group, consistent with findings in pigs exposed to ZEA (40 μg/kg BW) + DON (12 μg/kg BW) (Piotrowska et al., 2014). These shifts suggest that synbiotic supplementation may help suppress opportunistic pathogens while promoting beneficial taxa involved in gut health and mycotoxin detoxification.

On d21, FUM + DON markedly increased the relative abundance of *Clostridiaceae* by 17-fold, whereas synbiotic supplementation in the FUM + DON treatment group decreased the relative abundance by 10.5-fold, suggesting a protective effect against necrotic enteritis, which is often associated with *Clostridium perfringens* proliferation under mycotoxin challenge (Antonissen et al., 2015a; Shanmugasundaram et al., 2023). Similarly, on d21, the relative abundance of *Dehalobacteriaceae* and *Enterococaceae* showed numerical increase in the FUM + DON group, but synbiotic supplementation did not fully restore their relative abundance in the FUM + DON treatment group. The reduced *Enterococaceae* abundance, despite *E. faecium*, which belongs to the family *Enterococcaceae*, a synbiotic component, may indicate poor colonization or lack of direct enzymatic detoxification activity against FUM and DON.

On d35, the relative abundance of *Lactobacillaceae* was numerically increased by 2.7-fold in the FUM + DON treatment group, which contrasts with previous findings in chickens (Shanmugasundaram et al., 2023; Yu et al., 2022). A similar trend was observed in pigs when they were exposed to FB1 (12 mg/kg feed) alone for 29 days; fecal microbiota shifted toward higher levels of *Lactobacillus.* This increase likely reflects a compensatory shift following reductions in *Lachnospiraceae* and associated genera such as *Coprococcus* and *Gemmiger* (Mateos et al., 2018). *Lactobacillus* species are known for their ability to bind mycotoxins via cell wall components and prevent their absorption in the gut (Deepthi et al., 2016; Zhao et al., 2016). Further, the relative abundance of *Rikenellaceae* and *Bacteroidaceae*, key members of the Bacteroidetes phylum, numerically decreased in the FUM + DON group on d35, and was not reversed by synbiotic supplementation, indicating persistent intestinal dysbiosis because of the synergistic effect of combined mycotoxin challenge. These results contrast with previous studies in mice orally gavaged with DON alone (1 mg/kg or 5 mg/kg BW every 2 days for 14 days), increased *Bacteroidota* relative abundance (Wang et al., 2019). Given that *Bacteroidota* are considered potential contributors to gut health in chickens, their reduction most likely reflects intestinal dysbiosis induced by mycotoxin challenge. Mycotoxins induce microbiota shift, that influence the immune function of the host through microbial-derived metabolites, mainly SCFAs and activation of microbial-associated molecular patterns. Members of Lachnospiraceae and Ruminococcaceae produce SCFAs, which support gut integrity, prevent leaky gut and enhance mucosal immunity. The pen−based replication used in this study aligns with standard poultry research practices, though subtle microbial shifts may require larger sample sizes for confirmation. The main treatment related effects observed in this study were supported by consistent taxonomic, metabolic, and immunological patterns.

In summary, the trend toward lower bile FUM−specific IgA in birds exposed to FUM + DON suggests that even low−level mycotoxin contamination may impair local mucosal immunity. Such subtle immune alterations would not be easily detected under commercial conditions but could reduce protective capacity against enteric pathogens or vaccination challenges. The absence of differences in serum FUM− and DON−specific IgY concentrations suggests that subclinical mycotoxin levels used in this study did not significantly suppress systemic humoral immunity, and synbiotics did not enhance systemic antibody production under these conditions. This pattern aligns with field observations where early immune changes typically occur in the gut before systemic effects become detectable. Synbiotic supplementation at 0.05% modulated the relative abundance of major bacterial phyla, including *Firmicutes, Bacteroidetes, and Proteobacteria*. Functional prediction analysis supported by shifts in SCFA profiles suggested that potential microbial metabolic shifts associated with changes in microbial composition occurred during combined mycotoxin exposure. Given that co-contamination with low dose mycotoxin is increasingly common in modern poultry operations due to variable grain quality and global ingredient sourcing, synbiotics may offer modest benefits by helping birds maintain gut health and better cope with enteric challenges. However, synbiotics should be considered a complementary tool within a broader mycotoxin−management strategy, not as a standalone solution. Future research should quantify mycotoxin metabolites within the gut lumen and cecal content, to better define *in vivo* detoxification mechanism. Further, *in vitro* studies with dose-dependent interactions between synbiotic and FUM + DON, along with metabolomic profiling of culture supernatant, would further provide microbial detoxification pathways and functional metabolite production.

## Data Availability

The sequencing data generated in this study have been deposited in the NCBI Sequence Read Archive (SRA) under BioProject accession number PRJNA1438226.
