# Supplementary figures and images for: Synbiotic supplementation modulates humoral immunity and cecal microbiota in broiler chickens exposed to subclinical doses of fumonisins and deoxynivalenol
Source: Front Physiol. 2026 Apr 21;17:1808482. doi: 10.3389/fphys.2026.1808482 (PMC13138969; doi:10.3389/fphys.2026.1808482)

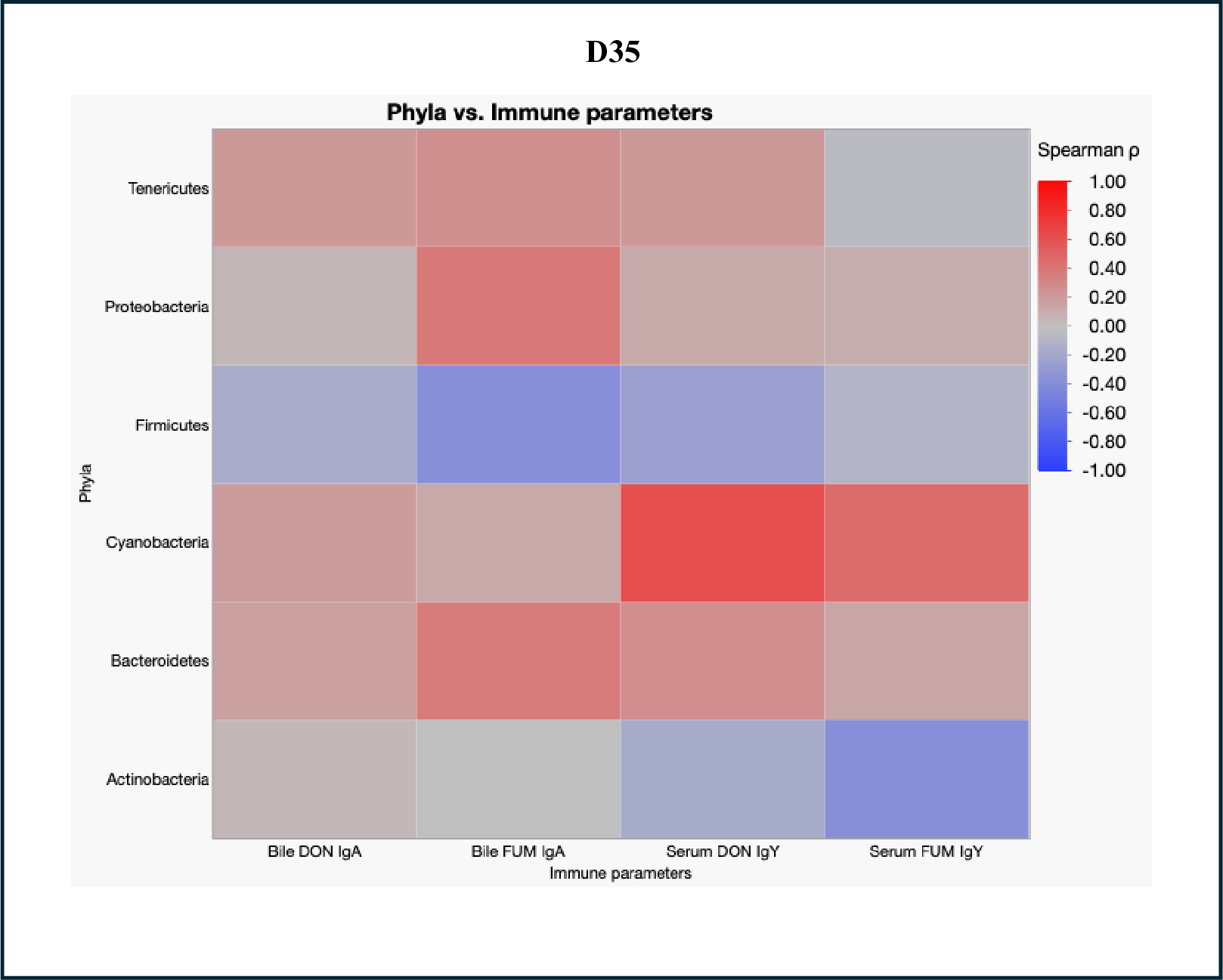

Supplement: Supplementary file 1 [file Image1.tif]
